# Exploring the causal impact of body mass index on metabolic biomarkers and cholelithiasis risk: a Mendelian randomization analysis

**DOI:** 10.1038/s41598-024-83217-6

**Published:** 2025-01-02

**Authors:** Feng Zhao, Yanjiang Yang, Wenwen Yang

**Affiliations:** 1 The First Hospital of Anhui University of Science & Technology (Huainan First People’s Hospital), Huainan, 232000 Anhui Province China; 2 Department of Rheumatology and Immunology, The people’s Hospital of Qiandongnan Autonomous Prefecture, Kaili, 556000 Guizhou Province China; 3 The First Clinical Medical College, Lanzhou University, Lanzhou, 730000 Gansu Province China

**Keywords:** Body Mass Index, Metabolic Biomarkers, Cholelithiasis, Mendelian Randomization, Liver diseases, Gall bladder disease, Obesity, Risk factors, Epidemiology

## Abstract

Obesity is a well-established risk factor for various diseases, but the mechanisms through which it influences disease development remain unclear. Using Mendelian randomization (MR) analysis, we examined the causal relationship between BMI, 249 metabolic traits, and cholelithiasis. BMI data were obtained from four sources, and cholelithiasis data were from two distinct datasets. We analyzed the direct effect of BMI on cholelithiasis and identified key metabolic mediators. BMI was found to be positively associated with the risk of cholelithiasis across all datasets analyzed. A total of 176 metabolites were identified to be significantly associated with BMI, including amino acids, cholesterol esters, free cholesterol, triglycerides, and phospholipids. Among these, 49 metabolites were identified as mediators in the BMI-cholelithiasis relationship. Specifically, fatty acid levels, cholesteryl esters, phospholipids, triglycerides, and free cholesterol were key mediators in this relationship, with mediation proportions ranging from − 2.38–7.14%. This study provides robust evidence that BMI significantly impacts metabolic biomarkers, which in turn affect the risk of cholelithiasis. These findings highlight the importance of managing BMI to mitigate metabolic dysfunction and reduce the risk of gallstone formation. Future research should explore the specific metabolic pathways involved to identify potential therapeutic targets.

**Supplementary Information:**

The online version contains supplementary material available at 10.1038/s41598-024-83217-6.

## Introduction

According to the World Health Organization, in 2022, global obesity rates continued to rise, with 1 in 8 people living with obesity, as adult obesity more than doubled and adolescent obesity quadrupled since 1990, while 2.5 billion adults were overweight, including 890 million with obesity^1^. It is widely recognized that obesity is a risk factor for many diseases^2–5^; however, the mechanisms through which obesity influences disease development are not yet fully understood^6,7^. Metabolic biomarkers play a significant role in understanding the underlying mechanisms through which Body mass index (BMI) contributes to disease risk, particularly in the context of lipid metabolism and other metabolic pathways. Previous studies have established the link between BMI and certain metabolic traits, such as amino acids^8,9^ and cholesterol levels^10^, it remains unclear whether BMI can influence the risk of various diseases by affecting metabolite levels. BMI is a key indicator of obesity and has been widely associated with various metabolic disorders, including cholelithiasis (gallstone formation)^11^. Therefore, this study aims to explore whether BMI influences metabolite levels and, in turn, affects disease risk, specifically examining whether changes in BMI lead to alterations in blood metabolite profiles that contribute to the risk of developing cholelithiasis employing Mendelian randomization (MR) analysis. MR offers a robust approach to exploring the causal relationships between BMI, metabolic biomarkers, and cholelithiasis, as it minimizes confounding factors by using genetic variants as instrumental variables (IVs)^12^. This study’s exploration of BMI’s influence on metabolic biomarkers and its causal relationship with cholelithiasis could provide critical insights into the mechanisms driving obesity-related diseases. By identifying key metabolic mediators, this research could inform future therapeutic interventions aimed at mitigating the metabolic dysfunctions associated with elevated BMI and reducing the risk of gallstone formation and other obesity-related conditions.

## Methods

**Fig. 1:**
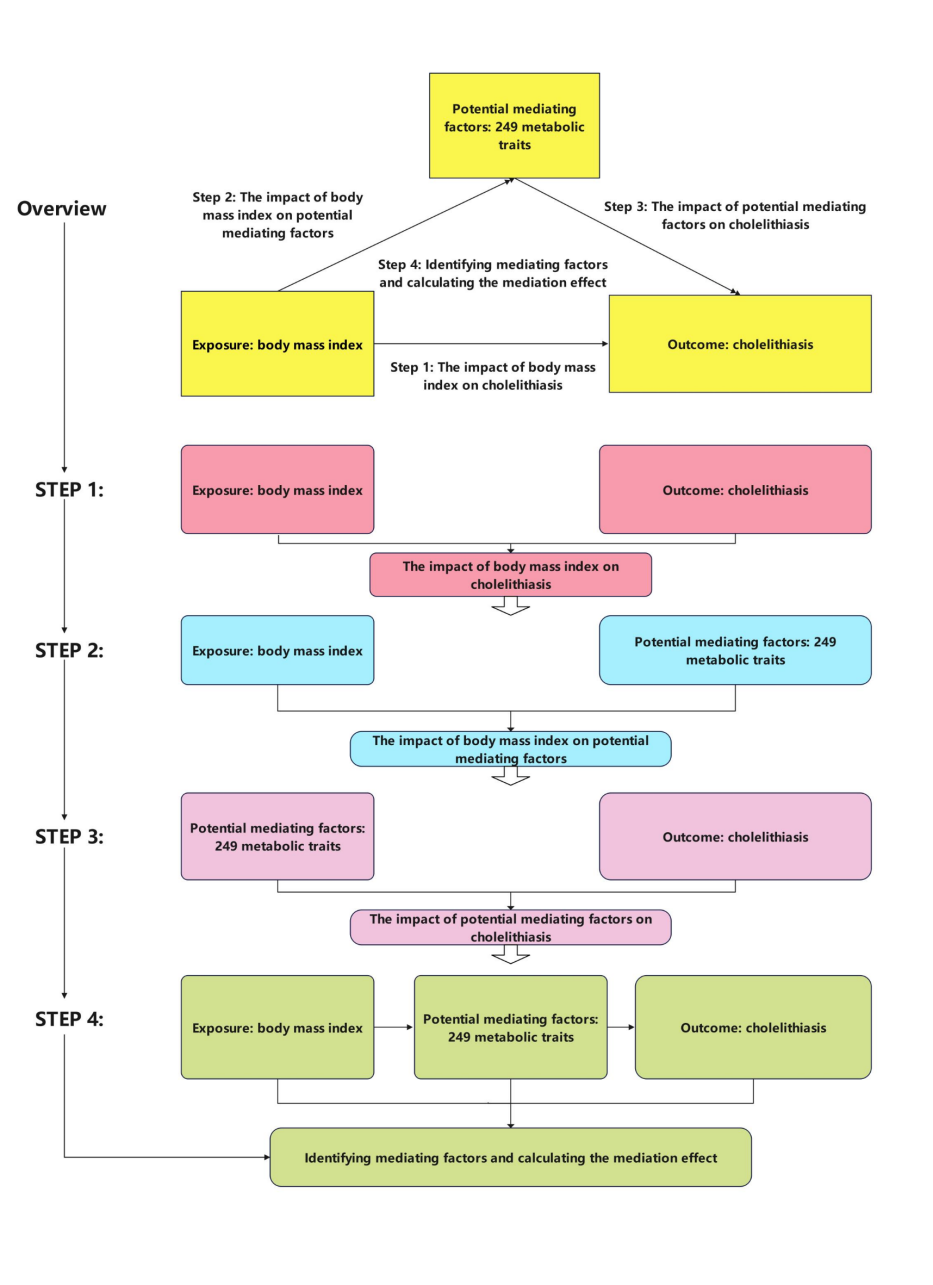
Analysis process flowchart. The analytical process of this study consists of 4 key steps, as depicted in the figure. Step 1 examines the impact of body mass index (BMI) on cholelithiasis (gallstones). Step 2 investigates the relationship between BMI and potential mediating factors (249 metabolic traits).Step 3 explores how these potential mediating factors influence cholelithiasis. Step 4 identifies the mediating factors and calculates the proportion of the mediating effect relative to the total effect.

The analytical process of this study involves four key steps, as illustrated in Fig. 1. First, the direct effect of BMI on cholelithiasis is examined. Next, the relationship between BMI and 249 metabolic traits, considered potential mediating factors, is explored. The third step assesses how these metabolic traits influence cholelithiasis, evaluating their role as mediators. Finally, the fourth step identifies the significant mediators and calculates the proportion of the mediating effect relative to the total effect of BMI on cholelithiasis. This comprehensive approach sheds light on the pathways through which BMI influences cholelithiasis risk via metabolic mediators.

### Data sources

As presented in Table 1, this study incorporated BMI data from four different sources, 249 blood metabolites^13^, and cholelithiasis data from two distinct sources. To minimize sample overlap, BMI from the GIANT consortium (id: ieu-a-835) was chosen as the exposure, and cholelithiasis from the Finngen Biobank as the outcome, to examine whether BMI is associated with cholelithiasis and whether it influences cholelithiasis risk through its effects on blood metabolites. All data used in this study are available from the IEU OpenGWAS project^14^ and the FinnGen Biobank^15^. Since the MR analysis used publicly available data from open-access databases and involved no individual-level data, this research did not require ethics committee approval.

**Table 1:** Information of the outcomes, mediating factors, and exposures.

		Phenocode in IEU OpenGWAS project or Finngen Biobank.	Sample size	The consortium or biobank of the data source	Number of European-descent cases	Number of European-descent controls
Exposure	Body mass index	ieu-b-40	681,275	GIANT consortium	NA	NA
		NA	290,820	Finngen Biobank’s 10th Round (R10) Release	NA	NA
		ieu-b-4816	99,998	Within family GWAS consortium	NA	NA
		ieu-a-835	322,154	GIANT consortium	NA	NA
Mediating factors	249 metabolic traits	ebi-a-GCST90092803 - ebi-a-GCST90093051	110,051-115082	The UK Biobank	NA	NA
Otcome	Cholelithiasis	ukb-a-559	337,199	The UK Biobank	6,986	330,213
		K11_CHOLELITH	401,832	Finngen Biobank’s 10th Round (R10) Release	40,191	361,641

*IEU* Integrative Epidemiology Unit, *GWAS* Genome-Wide Association Studies; *GIANT* the Genetic Investigation of ANthropometric Traits. More information about exposure, mediating factors, and outcomes is available at the IEU OpenGWAS project (https://gwas.mrcieu.ac.uk/) and the Finngen Biobank (https://r10.risteys.finngen.fi/).BMI data from the Finngen Biobank is available for download in the latest release (https://www.finngen.fi/en/access_results).

### Criteria for choosing IVs

In MR study, IVs are used to determine causal relationships between an exposure and an outcome by minimizing confounding and avoiding reverse causality. IVs, typically single nucleotide polymorphisms (SNPs), act as proxies for the exposure, helping to infer causality in a manner analogous to randomized controlled trials^16^. SNP selection was guided by several criteria, including a clumping window of 10,000 kb to account for physical proximity, strong linkage disequilibrium (r^2^ < 0.001), and a genome-wide significance threshold (*p* < 5 × 10^− 8^) to ensure robust associations with the exposure of interest. In all analyses, SNPs significantly associated with the outcome (*p* < 5 × 10^− 8^) were excluded to minimize bias and ensure adherence to the assumptions of Mendelian Randomization.

### Statistical analysis

In this study, the inverse-variance weighted (IVW) approach is regarded as the primary method for determining causal relationships due to its superior performance compared to the other two methods (Weighted Median and MR-Egger method). To ensure the IVW method operates correctly, horizontal pleiotropy must be either balanced or negligible^17^. The MR-Egger method, which accommodates non-zero intercepts, was applied to evaluate horizontal pleiotropy. We applied false discovery rate (FDR)^18^ adjustments to lower the FDR during the multiple hypothesis testing procedure. Cochran’s Q-test was applied to assess heterogeneity; if the p-value was below 0.05, a random-effects IVW model was implemented, whereas a fixed-effects IVW model was applied if the p-value exceeded 0.05. The MR-PRESSO technique was implemented to detect outliers. All statistical analyses were conducted with R software (version 4.3.2) and the TwoSampleMR package^19^ (version 0.5.10).

### Mediation analysis

Utilizing 249 metabolites as possible mediators, we conducted a two-step mediation MR analysis to investigate whether BMI-induced changes in blood metabolites could influence the risk of cholelithiasis. In the first step, we analyzed the relationship between BMI and 249 metabolic traits (β1). In the second step, we explored the association between these metabolic traits and cholelithiasis (β2). From the results, we identified potential mediators and calculated the mediation effect (β1*β2) as a proportion of the total effect of BMI on cholelithiasis.

## Results

This study initially analyzed the impact of BMI from four different sources on cholelithiasis from two distinct datasets, with the results detailed in Supplementary Tables 1, 2. Across all analyses, BMI was consistently found to be positively associated with the risk of cholelithiasis. Subsequently, we evaluated the effects of BMI from four distinct sources on 249 metabolites, with the results detailed in Supplementary Tables 3–6. As shown in Supplementary Tables 7, to assess whether BMI from different sources exerted similar effects on metabolite levels, we identified 159 metabolites that were associated with all four BMI sources based on FDR-adjusted q-values. Through pairwise comparisons of their β-values, we observed that BMI exhibited consistent effects on these metabolites, with their levels either increasing or decreasing in response to higher BMI. Subsequently, we examined the effects of 249 metabolic traits on cholelithiasis from two different sources, with the results detailed in Supplementary Tables 8–9. To evaluate the impact of these metabolites on cholelithiasis across sources, we identified 54 metabolites associated with both cholelithiasis sources based on FDR-adjusted q-values, with the results shown in Supplementary Table 10. Pairwise comparisons of their β-values revealed that these metabolites consistently influenced cholelithiasis, with risk either increasing or decreasing in response to changes in metabolite concentrations. Based on a comparison of the relationships between BMI from four different sources, cholelithiasis from two different sources, and metabolites, we observed a high degree of consistency in the effects of BMI on metabolites as well as the effects of metabolites on cholelithiasis. To minimize sample overlap between the exposure, mediators, and outcomes, we selected BMI from the GIANT consortium (id: ieu-a-835) as the exposure and cholelithiasis from the Finngen Biobank as the outcome for subsequent analysis. In Table 2, we identified 176 metabolites associated with BMI from the GIANT consortium based on FDR-adjusted q-values. In Table 3, we identified 85 metabolites associated with cholelithiasis from FinnGen. In Table 4, we present 49 metabolites identified as mediators—BMI may influence the risk of cholelithiasis by affecting these metabolites. We categorized these metabolites into 10 groups, with the classification details available in Table S4 of the original publication^13^ on the 249 metabolites.

**Table 2:** The metabolic traits associated with body mass index, filtered based on the q-values obtained from the IVW method, with the exclusion of metabolic traits exhibiting horizontal pleiotropy.

Categories	Serial numbers	Outcome	exposure	Number of SNPs	IVW_Beta	IVW_se	IVW_pval	IVW_q_value	egger_intercept	se	pval
Amino acids	1	Phenylalanine levels || id: ebi-a-GCST90092936	Body mass index || id: ieu-a-835	68	1.16E-01	2.32E-02	6.56E-07	7.10E-06	4.74E-04	1.88E-03	8.02E-01
	2	Glutamine levels || id: ebi-a-GCST90092818	Body mass index || id: ieu-a-835	67	-1.00E-01	3.30E-02	2.37E-03	4.22E-03	-4.67E-03	2.60E-03	7.67E-02
	3	Glycine levels || id: ebi-a-GCST90092820	Body mass index || id: ieu-a-835	67	-1.36E-01	2.50E-02	5.35E-08	7.01E-07	-9.96E-04	2.02E-03	6.24E-01
	4	Isoleucine levels || id: ebi-a-GCST90092843	Body mass index || id: ieu-a-835	68	1.18E-01	2.31E-02	3.18E-07	3.95E-06	8.47E-04	1.87E-03	6.51E-01
	5	Total concentration of branched-chain amino acids (leucine + isoleucine + valine) || id: ebi-a-GCST90092984	Body mass index || id: ieu-a-835	68	1.50E-01	2.52E-02	2.48E-09	6.69E-08	2.30E-04	2.03E-03	9.10E-01
	6	Leucine levels || id: ebi-a-GCST90092891	Body mass index || id: ieu-a-835	68	1.25E-01	2.48E-02	4.72E-07	5.60E-06	-1.47E-04	2.01E-03	9.42E-01
	7	Tyrosine levels || id: ebi-a-GCST90092993	Body mass index || id: ieu-a-835	68	1.53E-01	2.62E-02	5.49E-09	1.05E-07	1.85E-04	2.12E-03	9.30E-01
	8	Valine levels || id: ebi-a-GCST90092995	Body mass index || id: ieu-a-835	68	1.66E-01	2.60E-02	1.89E-10	2.35E-08	1.58E-04	2.10E-03	9.40E-01
Cholesteryl ester levels	9	Cholesteryl ester levels in HDL || id: ebi-a-GCST90092823	Body mass index || id: ieu-a-835	63	-1.60E-01	3.85E-02	3.06E-05	1.23E-04	-4.32E-03	3.10E-03	1.69E-01
	10	Cholesteryl ester levels in IDL || id: ebi-a-GCST90092833	Body mass index || id: ieu-a-835	67	-1.14E-01	3.40E-02	7.85E-04	1.69E-03	1.44E-03	2.72E-03	5.99E-01
	11	Cholesteryl esters to total lipids ratio in IDL || id: ebi-a-GCST90092834	Body mass index || id: ieu-a-835	66	-1.31E-01	3.13E-02	2.79E-05	1.14E-04	1.68E-04	2.52E-03	9.47E-01
	12	Cholesteryl ester levels in large HDL || id: ebi-a-GCST90092846	Body mass index || id: ieu-a-835	63	-2.22E-01	3.62E-02	9.53E-10	5.03E-08	-1.72E-03	2.88E-03	5.52E-01
	13	Cholesteryl esters to total lipids ratio in large HDL || id: ebi-a-GCST90092847	Body mass index || id: ieu-a-835	65	-2.00E-01	4.02E-02	6.53E-07	7.10E-06	-5.08E-03	3.14E-03	1.11E-01
	14	Cholesteryl ester levels in large LDL || id: ebi-a-GCST90092858	Body mass index || id: ieu-a-835	67	-8.00E-02	3.20E-02	1.25E-02	1.92E-02	3.79E-04	2.57E-03	8.83E-01
	15	Cholesteryl esters to total lipids ratio in large LDL || id: ebi-a-GCST90092859	Body mass index || id: ieu-a-835	68	-8.54E-02	2.94E-02	3.67E-03	6.17E-03	-8.40E-04	2.38E-03	7.25E-01
	16	Cholesteryl esters to total lipids ratio in large VLDL || id: ebi-a-GCST90092871	Body mass index || id: ieu-a-835	67	-9.80E-02	3.01E-02	1.11E-03	2.19E-03	-3.11E-03	2.38E-03	1.96E-01
	17	Cholesteryl ester levels in medium HDL || id: ebi-a-GCST90092894	Body mass index || id: ieu-a-835	66	-1.57E-01	3.57E-02	1.13E-05	5.21E-05	-1.65E-03	2.86E-03	5.66E-01
	18	Cholesteryl esters to total lipids ratio in medium HDL || id: ebi-a-GCST90092895	Body mass index || id: ieu-a-835	67	-1.59E-01	3.71E-02	1.96E-05	8.41E-05	-4.61E-03	2.95E-03	1.24E-01
	19	Cholesteryl ester levels in medium VLDL || id: ebi-a-GCST90092918	Body mass index || id: ieu-a-835	67	-7.56E-02	2.87E-02	8.33E-03	1.31E-02	3.57E-04	2.30E-03	8.77E-01
	20	Cholesteryl esters to total lipids ratio in medium VLDL || id: ebi-a-GCST90092919	Body mass index || id: ieu-a-835	67	-1.31E-01	3.55E-02	2.38E-04	6.16E-04	-3.51E-03	2.82E-03	2.17E-01
	21	Cholesteryl esters to total lipids ratio in small HDL || id: ebi-a-GCST90092947	Body mass index || id: ieu-a-835	68	-6.18E-02	2.73E-02	2.38E-02	3.44E-02	-1.76E-03	2.20E-03	4.27E-01
	22	Cholesteryl ester levels in very large HDL || id: ebi-a-GCST90093006	Body mass index || id: ieu-a-835	64	-2.25E-01	3.79E-02	2.96E-09	6.69E-08	-2.79E-03	3.00E-03	3.57E-01
	23	Cholesteryl esters to total lipids ratio in very large HDL || id: ebi-a-GCST90093007	Body mass index || id: ieu-a-835	68	-1.17E-01	3.52E-02	9.10E-04	1.89E-03	-1.95E-04	2.84E-03	9.45E-01
	24	Cholesteryl ester levels in very large VLDL || id: ebi-a-GCST90093018	Body mass index || id: ieu-a-835	68	7.74E-02	2.84E-02	6.44E-03	1.04E-02	1.77E-03	2.29E-03	4.42E-01
	25	Cholesteryl esters to total lipids ratio in very large VLDL || id: ebi-a-GCST90093019	Body mass index || id: ieu-a-835	68	-1.36E-01	3.36E-02	5.37E-05	1.91E-04	-3.89E-03	2.67E-03	1.50E-01
	26	Cholesteryl ester levels in very small VLDL || id: ebi-a-GCST90093030	Body mass index || id: ieu-a-835	67	-8.76E-02	2.91E-02	2.57E-03	4.51E-03	7.65E-04	2.33E-03	7.44E-01
	27	Cholesteryl esters to total lipids ratio in very small VLDL || id: ebi-a-GCST90093031	Body mass index || id: ieu-a-835	66	-1.63E-01	3.42E-02	1.89E-06	1.43E-05	-3.39E-03	2.72E-03	2.17E-01
	28	Cholesteryl ester levels in chylomicrons and extremely large VLDL || id: ebi-a-GCST90093042	Body mass index || id: ieu-a-835	67	8.32E-02	2.78E-02	2.79E-03	4.83E-03	3.24E-03	2.22E-03	1.49E-01
	29	Cholesteryl esters to total lipids ratio in chylomicrons and extremely large VLDL || id: ebi-a-GCST90093043	Body mass index || id: ieu-a-835	68	-8.70E-02	2.48E-02	4.51E-04	1.06E-03	-1.84E-03	1.99E-03	3.59E-01
Free cholesterol levels	30	Free cholesterol levels in HDL || id: ebi-a-GCST90092824	Body mass index || id: ieu-a-835	64	-1.65E-01	3.62E-02	5.13E-06	2.84E-05	-2.03E-03	2.87E-03	4.82E-01
	31	Free cholesterol levels in IDL || id: ebi-a-GCST90092835	Body mass index || id: ieu-a-835	67	-1.04E-01	3.31E-02	1.66E-03	3.05E-03	2.15E-04	2.65E-03	9.36E-01
	32	Free cholesterol to total lipids ratio in IDL || id: ebi-a-GCST90092836	Body mass index || id: ieu-a-835	68	-7.94E-02	2.96E-02	7.23E-03	1.15E-02	-4.10E-03	2.34E-03	8.42E-02
	33	Free cholesterol levels in large HDL || id: ebi-a-GCST90092848	Body mass index || id: ieu-a-835	64	-2.13E-01	3.59E-02	2.76E-09	6.69E-08	-2.20E-03	2.85E-03	4.43E-01
	34	Free cholesterol to total lipids ratio in large HDL || id: ebi-a-GCST90092849	Body mass index || id: ieu-a-835	67	-1.47E-01	3.87E-02	1.54E-04	4.21E-04	-3.01E-03	3.09E-03	3.33E-01
	35	Free cholesterol levels in large LDL || id: ebi-a-GCST90092860	Body mass index || id: ieu-a-835	67	-1.09E-01	3.39E-02	1.33E-03	2.57E-03	1.74E-04	2.72E-03	9.49E-01
	36	Free cholesterol to total lipids ratio in large LDL || id: ebi-a-GCST90092861	Body mass index || id: ieu-a-835	66	-1.53E-01	3.38E-02	6.06E-06	3.28E-05	-4.31E-03	2.66E-03	1.10E-01
	37	Free cholesterol levels in large VLDL || id: ebi-a-GCST90092872	Body mass index || id: ieu-a-835	68	1.09E-01	2.98E-02	2.53E-04	6.49E-04	2.18E-03	2.39E-03	3.65E-01
	38	Free cholesterol to total lipids ratio in large VLDL || id: ebi-a-GCST90092873	Body mass index || id: ieu-a-835	68	9.80E-02	2.83E-02	5.27E-04	1.19E-03	-3.55E-04	2.28E-03	8.77E-01
	39	Free cholesterol levels in LDL || id: ebi-a-GCST90092885	Body mass index || id: ieu-a-835	67	-9.04E-02	3.29E-02	6.01E-03	9.84E-03	1.91E-04	2.64E-03	9.43E-01
	40	Free cholesterol levels in medium HDL || id: ebi-a-GCST90092896	Body mass index || id: ieu-a-835	66	-1.56E-01	3.52E-02	9.46E-06	4.71E-05	-1.61E-03	2.82E-03	5.70E-01
	41	Free cholesterol to total lipids ratio in medium HDL || id: ebi-a-GCST90092897	Body mass index || id: ieu-a-835	64	-1.65E-01	3.84E-02	1.77E-05	7.72E-05	-2.55E-03	3.04E-03	4.04E-01
	42	Free cholesterol to total lipids ratio in medium LDL || id: ebi-a-GCST90092909	Body mass index || id: ieu-a-835	67	-1.34E-01	3.51E-02	1.33E-04	3.78E-04	-3.88E-03	2.80E-03	1.70E-01
	43	Free cholesterol to total lipids ratio in medium VLDL || id: ebi-a-GCST90092921	Body mass index || id: ieu-a-835	67	-1.14E-01	3.36E-02	6.84E-04	1.49E-03	-3.20E-03	2.67E-03	2.35E-01
	44	Free cholesterol levels in small HDL || id: ebi-a-GCST90092948	Body mass index || id: ieu-a-835	67	-8.02E-02	3.28E-02	1.44E-02	2.17E-02	1.87E-03	2.62E-03	4.79E-01
	45	Free cholesterol to total lipids ratio in small HDL || id: ebi-a-GCST90092949	Body mass index || id: ieu-a-835	67	-1.50E-01	3.88E-02	1.15E-04	3.42E-04	-3.32E-03	3.09E-03	2.86E-01
	46	Free cholesterol to total lipids ratio in small LDL || id: ebi-a-GCST90092961	Body mass index || id: ieu-a-835	68	-1.13E-01	3.32E-02	6.30E-04	1.40E-03	-3.26E-03	2.65E-03	2.23E-01
	47	Free cholesterol to total lipids ratio in small VLDL || id: ebi-a-GCST90092973	Body mass index || id: ieu-a-835	66	-1.50E-01	3.19E-02	2.60E-06	1.75E-05	-1.84E-03	2.54E-03	4.70E-01
	48	Total free cholesterol levels || id: ebi-a-GCST90092988	Body mass index || id: ieu-a-835	67	-1.03E-01	3.25E-02	1.49E-03	2.77E-03	5.79E-04	2.61E-03	8.25E-01
	49	Free cholesterol levels in very large HDL || id: ebi-a-GCST90093008	Body mass index || id: ieu-a-835	66	-1.70E-01	3.82E-02	8.32E-06	4.32E-05	-4.04E-03	3.03E-03	1.88E-01
	50	Free cholesterol to total lipids ratio in very large HDL || id: ebi-a-GCST90093009	Body mass index || id: ieu-a-835	64	2.18E-01	3.27E-02	2.46E-11	6.13E-09	2.87E-03	2.58E-03	2.71E-01
	51	Free cholesterol levels in very large VLDL || id: ebi-a-GCST90093020	Body mass index || id: ieu-a-835	68	1.08E-01	2.95E-02	2.34E-04	6.16E-04	2.12E-03	2.37E-03	3.73E-01
	52	Free cholesterol to total lipids ratio in very large VLDL || id: ebi-a-GCST90093021	Body mass index || id: ieu-a-835	67	-9.08E-02	2.85E-02	1.45E-03	2.72E-03	-4.00E-03	2.23E-03	7.79E-02
	53	Free cholesterol to total lipids ratio in very small VLDL || id: ebi-a-GCST90093033	Body mass index || id: ieu-a-835	67	-7.22E-02	2.65E-02	6.51E-03	1.05E-02	-2.68E-03	2.10E-03	2.08E-01
	54	Free cholesterol levels in chylomicrons and extremely large VLDL || id: ebi-a-GCST90093044	Body mass index || id: ieu-a-835	68	1.21E-01	3.05E-02	7.58E-05	2.52E-04	2.60E-03	2.44E-03	2.91E-01
	55	Free cholesterol to total lipids ratio in chylomicrons and extremely large VLDL || id: ebi-a-GCST90093045	Body mass index || id: ieu-a-835	68	-6.19E-02	2.23E-02	5.46E-03	9.00E-03	-1.13E-03	1.80E-03	5.32E-01
Cholesterol levels	56	Clinical LDL cholesterol levels || id: ebi-a-GCST90092814	Body mass index || id: ieu-a-835	67	-6.79E-02	3.05E-02	2.60E-02	3.72E-02	6.12E-04	2.45E-03	8.03E-01
	57	HDL cholesterol levels || id: ebi-a-GCST90092822	Body mass index || id: ieu-a-835	64	-1.76E-01	3.77E-02	3.01E-06	1.92E-05	-2.11E-03	2.99E-03	4.84E-01
	58	Cholesterol levels in IDL || id: ebi-a-GCST90092831	Body mass index || id: ieu-a-835	67	-1.12E-01	3.38E-02	9.20E-04	1.89E-03	1.13E-03	2.71E-03	6.78E-01
	59	Cholesterol to total lipids ratio in IDL || id: ebi-a-GCST90092832	Body mass index || id: ieu-a-835	66	-1.30E-01	3.24E-02	5.78E-05	2.00E-04	-1.97E-03	2.59E-03	4.50E-01
	60	Cholesterol levels in large HDL || id: ebi-a-GCST90092844	Body mass index || id: ieu-a-835	63	-2.19E-01	3.60E-02	1.24E-09	5.14E-08	-1.72E-03	2.86E-03	5.50E-01
	61	Cholesterol to total lipids ratio in large HDL || id: ebi-a-GCST90092845	Body mass index || id: ieu-a-835	65	-1.96E-01	4.14E-02	2.13E-06	1.52E-05	-5.22E-03	3.24E-03	1.12E-01
	62	Cholesterol levels in large LDL || id: ebi-a-GCST90092856	Body mass index || id: ieu-a-835	67	-8.86E-02	3.26E-02	6.57E-03	1.05E-02	3.17E-04	2.62E-03	9.04E-01
	63	Cholesterol to total lipids ratio in large LDL || id: ebi-a-GCST90092857	Body mass index || id: ieu-a-835	66	-1.71E-01	3.69E-02	3.46E-06	2.10E-05	-3.28E-03	2.94E-03	2.69E-01
	64	Cholesterol levels in large VLDL || id: ebi-a-GCST90092868	Body mass index || id: ieu-a-835	68	8.46E-02	2.86E-02	3.12E-03	5.33E-03	1.94E-03	2.30E-03	4.02E-01
	65	LDL cholesterol levels || id: ebi-a-GCST90092883	Body mass index || id: ieu-a-835	67	-6.55E-02	3.07E-02	3.28E-02	4.64E-02	4.06E-04	2.46E-03	8.70E-01
	66	Cholesterol levels in medium HDL || id: ebi-a-GCST90092892	Body mass index || id: ieu-a-835	66	-1.58E-01	3.57E-02	1.02E-05	4.97E-05	-1.65E-03	2.86E-03	5.67E-01
	67	Cholesterol to total lipids ratio in medium HDL || id: ebi-a-GCST90092893	Body mass index || id: ieu-a-835	66	-1.73E-01	4.02E-02	1.72E-05	7.67E-05	-4.67E-03	3.18E-03	1.47E-01
	68	Cholesterol to total lipids ratio in medium LDL || id: ebi-a-GCST90092905	Body mass index || id: ieu-a-835	66	-1.45E-01	2.57E-02	1.80E-08	2.64E-07	-2.64E-03	2.04E-03	2.01E-01
	69	Cholesterol to total lipids ratio in medium VLDL || id: ebi-a-GCST90092917	Body mass index || id: ieu-a-835	67	-1.27E-01	3.52E-02	3.16E-04	7.87E-04	-3.49E-03	2.79E-03	2.15E-01
	70	Cholesterol to total lipids ratio in small HDL || id: ebi-a-GCST90092945	Body mass index || id: ieu-a-835	68	-1.05E-01	3.31E-02	1.42E-03	2.69E-03	-2.89E-03	2.65E-03	2.80E-01
	71	Cholesterol to total lipids ratio in small LDL || id: ebi-a-GCST90092957	Body mass index || id: ieu-a-835	68	-1.24E-01	2.56E-02	1.33E-06	1.15E-05	-4.10E-04	2.07E-03	8.43E-01
	72	Cholesterol to total lipids ratio in small VLDL || id: ebi-a-GCST90092969	Body mass index || id: ieu-a-835	67	-7.77E-02	3.04E-02	1.07E-02	1.66E-02	-2.27E-03	2.43E-03	3.53E-01
	73	Total cholesterol levels || id: ebi-a-GCST90092985	Body mass index || id: ieu-a-835	66	-1.13E-01	3.37E-02	8.26E-04	1.74E-03	8.02E-04	2.68E-03	7.66E-01
	74	Total esterified cholesterol levels || id: ebi-a-GCST90092986	Body mass index || id: ieu-a-835	65	-1.08E-01	3.32E-02	1.16E-03	2.27E-03	-3.31E-04	2.64E-03	9.01E-01
	75	Cholesterol levels in very large HDL || id: ebi-a-GCST90093004	Body mass index || id: ieu-a-835	65	-1.95E-01	4.06E-02	1.59E-06	1.32E-05	-4.48E-03	3.19E-03	1.65E-01
	76	Cholesterol to total lipids ratio in very large HDL || id: ebi-a-GCST90093005	Body mass index || id: ieu-a-835	68	9.48E-02	2.76E-02	6.02E-04	1.35E-03	2.38E-03	2.21E-03	2.87E-01
	77	Cholesterol levels in very large VLDL || id: ebi-a-GCST90093016	Body mass index || id: ieu-a-835	68	9.43E-02	2.89E-02	1.11E-03	2.19E-03	1.91E-03	2.33E-03	4.16E-01
	78	Cholesterol to total lipids ratio in very large VLDL || id: ebi-a-GCST90093017	Body mass index || id: ieu-a-835	67	-1.23E-01	3.20E-02	1.25E-04	3.63E-04	-3.86E-03	2.52E-03	1.30E-01
	79	Cholesterol levels in very small VLDL || id: ebi-a-GCST90093028	Body mass index || id: ieu-a-835	67	-6.97E-02	2.79E-02	1.24E-02	1.91E-02	7.51E-04	2.24E-03	7.38E-01
	80	Cholesterol to total lipids ratio in very small VLDL || id: ebi-a-GCST90093029	Body mass index || id: ieu-a-835	66	-1.55E-01	3.38E-02	4.41E-06	2.50E-05	-3.49E-03	2.69E-03	1.98E-01
	81	Cholesterol levels in chylomicrons and extremely large VLDL || id: ebi-a-GCST90093040	Body mass index || id: ieu-a-835	68	1.09E-01	3.03E-02	3.20E-04	7.89E-04	2.39E-03	2.43E-03	3.29E-01
	82	Cholesterol to total lipids ratio in chylomicrons and extremely large VLDL || id: ebi-a-GCST90093041	Body mass index || id: ieu-a-835	68	-8.50E-02	2.36E-02	3.13E-04	7.87E-04	-1.21E-03	1.90E-03	5.26E-01
Particle concentrations or sizes	83	Concentration of HDL particles || id: ebi-a-GCST90092826	Body mass index || id: ieu-a-835	66	-1.46E-01	3.54E-02	3.96E-05	1.49E-04	-1.02E-03	2.84E-03	7.21E-01
	84	Concentration of large HDL particles || id: ebi-a-GCST90092851	Body mass index || id: ieu-a-835	63	-2.17E-01	3.52E-02	7.11E-10	5.03E-08	-1.68E-03	2.79E-03	5.49E-01
	85	Concentration of large VLDL particles || id: ebi-a-GCST90092875	Body mass index || id: ieu-a-835	68	1.05E-01	2.95E-02	3.56E-04	8.69E-04	2.25E-03	2.37E-03	3.47E-01
	86	Concentration of medium HDL particles || id: ebi-a-GCST90092899	Body mass index || id: ieu-a-835	66	-1.47E-01	3.35E-02	1.11E-05	5.21E-05	-1.32E-03	2.69E-03	6.26E-01
	87	Concentration of small VLDL particles || id: ebi-a-GCST90092975	Body mass index || id: ieu-a-835	68	6.19E-02	2.75E-02	2.45E-02	3.52E-02	1.44E-03	2.22E-03	5.19E-01
	88	Total concentration of lipoprotein particles || id: ebi-a-GCST90092990	Body mass index || id: ieu-a-835	66	-1.45E-01	3.59E-02	5.27E-05	1.91E-04	-8.56E-04	2.88E-03	7.68E-01
	89	Concentration of very large HDL particles || id: ebi-a-GCST90093011	Body mass index || id: ieu-a-835	65	-1.94E-01	3.98E-02	1.10E-06	1.01E-05	-4.67E-03	3.12E-03	1.39E-01
	90	Concentration of very large VLDL particles || id: ebi-a-GCST90093023	Body mass index || id: ieu-a-835	68	1.22E-01	3.08E-02	7.74E-05	2.54E-04	2.45E-03	2.48E-03	3.26E-01
	91	Concentration of chylomicrons and extremely large VLDL particles || id: ebi-a-GCST90093047	Body mass index || id: ieu-a-835	68	1.26E-01	3.07E-02	3.82E-05	1.46E-04	2.45E-03	2.46E-03	3.24E-01
	92	Average diameter for HDL particles || id: ebi-a-GCST90092828	Body mass index || id: ieu-a-835	65	-1.83E-01	3.78E-02	1.28E-06	1.13E-05	-4.09E-03	2.97E-03	1.73E-01
	93	Average diameter for LDL particles || id: ebi-a-GCST90092889	Body mass index || id: ieu-a-835	68	-8.60E-02	3.36E-02	1.05E-02	1.64E-02	1.75E-03	2.71E-03	5.19E-01
	94	Average diameter for VLDL particles || id: ebi-a-GCST90093002	Body mass index || id: ieu-a-835	68	1.32E-01	3.11E-02	2.23E-05	9.42E-05	2.16E-03	2.50E-03	3.90E-01
Triglyceride levels	95	Triglyceride levels in IDL || id: ebi-a-GCST90092841	Body mass index || id: ieu-a-835	68	7.71E-02	2.64E-02	3.51E-03	5.94E-03	1.71E-03	2.12E-03	4.23E-01
	96	Triglycerides to total lipids ratio in IDL || id: ebi-a-GCST90092842	Body mass index || id: ieu-a-835	66	1.47E-01	3.34E-02	1.11E-05	5.21E-05	3.10E-03	2.66E-03	2.48E-01
	97	Triglyceride levels in large HDL || id: ebi-a-GCST90092854	Body mass index || id: ieu-a-835	68	-9.15E-02	2.45E-02	1.91E-04	5.11E-04	4.37E-04	1.98E-03	8.26E-01
	98	Triglycerides to total lipids ratio in large HDL || id: ebi-a-GCST90092855	Body mass index || id: ieu-a-835	67	1.54E-01	3.95E-02	9.41E-05	3.00E-04	4.32E-03	3.16E-03	1.76E-01
	99	Triglyceride levels in large LDL || id: ebi-a-GCST90092866	Body mass index || id: ieu-a-835	68	7.86E-02	2.60E-02	2.53E-03	4.46E-03	1.30E-03	2.10E-03	5.38E-01
	100	Triglycerides to total lipids ratio in large LDL || id: ebi-a-GCST90092867	Body mass index || id: ieu-a-835	66	1.46E-01	3.18E-02	4.43E-06	2.50E-05	2.97E-03	2.53E-03	2.45E-01
	101	Triglyceride levels in large VLDL || id: ebi-a-GCST90092878	Body mass index || id: ieu-a-835	68	9.53E-02	2.91E-02	1.07E-03	2.15E-03	2.54E-03	2.34E-03	2.81E-01
	102	Triglyceride levels in LDL || id: ebi-a-GCST90092890	Body mass index || id: ieu-a-835	68	8.51E-02	2.66E-02	1.37E-03	2.63E-03	1.41E-03	2.14E-03	5.13E-01
	103	Triglycerides to total lipids ratio in medium HDL || id: ebi-a-GCST90092903	Body mass index || id: ieu-a-835	67	1.39E-01	3.73E-02	1.89E-04	5.11E-04	4.12E-03	2.98E-03	1.72E-01
	104	Triglyceride levels in medium LDL || id: ebi-a-GCST90092914	Body mass index || id: ieu-a-835	68	9.00E-02	2.70E-02	8.73E-04	1.83E-03	1.36E-03	2.18E-03	5.36E-01
	105	Triglycerides to total lipids ratio in medium LDL || id: ebi-a-GCST90092915	Body mass index || id: ieu-a-835	66	1.11E-01	2.91E-02	1.35E-04	3.78E-04	2.44E-03	2.32E-03	2.97E-01
	106	Triglyceride levels in medium VLDL || id: ebi-a-GCST90092926	Body mass index || id: ieu-a-835	68	7.70E-02	2.82E-02	6.32E-03	1.03E-02	2.35E-03	2.26E-03	3.02E-01
	107	Triglycerides to total lipids ratio in medium VLDL || id: ebi-a-GCST90092927	Body mass index || id: ieu-a-835	67	1.21E-01	3.46E-02	4.41E-04	1.05E-03	3.28E-03	2.74E-03	2.36E-01
	108	Triglyceride levels in small HDL || id: ebi-a-GCST90092954	Body mass index || id: ieu-a-835	68	1.32E-01	3.41E-02	1.03E-04	3.16E-04	3.19E-03	2.73E-03	2.47E-01
	109	Triglycerides to total lipids ratio in small HDL || id: ebi-a-GCST90092955	Body mass index || id: ieu-a-835	67	1.52E-01	3.62E-02	2.61E-05	1.08E-04	3.85E-03	2.89E-03	1.89E-01
	110	Triglyceride levels in small LDL || id: ebi-a-GCST90092966	Body mass index || id: ieu-a-835	68	9.36E-02	2.79E-02	8.07E-04	1.72E-03	1.92E-03	2.25E-03	3.96E-01
	111	Triglycerides to total lipids ratio in small LDL || id: ebi-a-GCST90092967	Body mass index || id: ieu-a-835	66	1.36E-01	2.91E-02	3.21E-06	2.00E-05	1.56E-03	2.31E-03	5.02E-01
	112	Triglyceride levels in small VLDL || id: ebi-a-GCST90092978	Body mass index || id: ieu-a-835	68	9.57E-02	2.96E-02	1.25E-03	2.42E-03	2.55E-03	2.38E-03	2.86E-01
	113	Triglycerides to total lipids ratio in small VLDL || id: ebi-a-GCST90092979	Body mass index || id: ieu-a-835	67	9.17E-02	3.16E-02	3.74E-03	6.26E-03	2.43E-03	2.52E-03	3.39E-01
	114	Ratio of triglycerides to phosphoglycerides || id: ebi-a-GCST90092983	Body mass index || id: ieu-a-835	67	1.58E-01	3.38E-02	2.88E-06	1.89E-05	3.89E-03	2.70E-03	1.54E-01
	115	Total triglycerides levels || id: ebi-a-GCST90092992	Body mass index || id: ieu-a-835	68	1.02E-01	2.93E-02	5.16E-04	1.18E-03	2.67E-03	2.34E-03	2.59E-01
	116	Triglyceride levels in VLDL || id: ebi-a-GCST90093003	Body mass index || id: ieu-a-835	68	1.05E-01	2.97E-02	3.86E-04	9.24E-04	2.67E-03	2.38E-03	2.65E-01
	117	Triglycerides to total lipids ratio in very large HDL || id: ebi-a-GCST90093015	Body mass index || id: ieu-a-835	67	1.59E-01	3.52E-02	6.40E-06	3.39E-05	4.74E-03	2.79E-03	9.45E-02
	118	Triglyceride levels in very large VLDL || id: ebi-a-GCST90093026	Body mass index || id: ieu-a-835	68	1.24E-01	3.16E-02	8.77E-05	2.84E-04	2.90E-03	2.53E-03	2.56E-01
	119	Triglycerides to total lipids ratio in very large VLDL || id: ebi-a-GCST90093027	Body mass index || id: ieu-a-835	67	9.83E-02	2.98E-02	9.74E-04	1.98E-03	4.00E-03	2.34E-03	9.24E-02
	120	Triglyceride levels in very small VLDL || id: ebi-a-GCST90093038	Body mass index || id: ieu-a-835	68	9.71E-02	2.86E-02	6.72E-04	1.48E-03	2.19E-03	2.29E-03	3.43E-01
	121	Triglycerides to total lipids ratio in very small VLDL || id: ebi-a-GCST90093039	Body mass index || id: ieu-a-835	66	1.33E-01	3.31E-02	5.58E-05	1.96E-04	3.19E-03	2.63E-03	2.30E-01
	122	Triglyceride levels in chylomicrons and extremely large VLDL || id: ebi-a-GCST90093050	Body mass index || id: ieu-a-835	68	1.18E-01	2.92E-02	5.32E-05	1.91E-04	2.52E-03	2.34E-03	2.85E-01
Phospholipid levels	123	Total cholines levels || id: ebi-a-GCST90092812	Body mass index || id: ieu-a-835	65	-1.14E-01	3.11E-02	2.56E-04	6.49E-04	3.94E-05	2.48E-03	9.87E-01
	124	Phosphatidylcholine levels || id: ebi-a-GCST90092937	Body mass index || id: ieu-a-835	65	-1.22E-01	3.06E-02	7.07E-05	2.41E-04	-7.00E-05	2.44E-03	9.77E-01
	125	Phosphoglycerides levels || id: ebi-a-GCST90092938	Body mass index || id: ieu-a-835	65	-9.48E-02	2.97E-02	1.40E-03	2.66E-03	4.37E-04	2.37E-03	8.54E-01
	126	Sphingomyelin levels || id: ebi-a-GCST90092982	Body mass index || id: ieu-a-835	65	-1.13E-01	3.42E-02	9.76E-04	1.98E-03	-3.56E-04	2.73E-03	8.97E-01
	127	Phospholipid levels in HDL || id: ebi-a-GCST90092827	Body mass index || id: ieu-a-835	66	-1.50E-01	3.39E-02	9.07E-06	4.61E-05	-1.64E-03	2.72E-03	5.48E-01
	128	Phospholipid levels in IDL || id: ebi-a-GCST90092839	Body mass index || id: ieu-a-835	67	-9.77E-02	3.20E-02	2.28E-03	4.11E-03	5.36E-04	2.57E-03	8.35E-01
	129	Phospholipid levels in large HDL || id: ebi-a-GCST90092852	Body mass index || id: ieu-a-835	64	-1.96E-01	3.41E-02	8.75E-09	1.56E-07	-1.57E-03	2.71E-03	5.65E-01
	130	Phospholipids to total lipids ratio in large HDL || id: ebi-a-GCST90092853	Body mass index || id: ieu-a-835	66	2.29E-01	3.83E-02	2.20E-09	6.69E-08	4.76E-03	2.99E-03	1.17E-01
	131	Phospholipid levels in large LDL || id: ebi-a-GCST90092864	Body mass index || id: ieu-a-835	67	-7.28E-02	3.07E-02	1.78E-02	2.61E-02	7.67E-04	2.46E-03	7.57E-01
	132	Phospholipid levels in large VLDL || id: ebi-a-GCST90092876	Body mass index || id: ieu-a-835	68	1.13E-01	3.07E-02	2.35E-04	6.16E-04	2.37E-03	2.46E-03	3.38E-01
	133	Phospholipids to total lipids ratio in large VLDL || id: ebi-a-GCST90092877	Body mass index || id: ieu-a-835	67	1.29E-01	3.36E-02	1.25E-04	3.63E-04	3.62E-03	2.68E-03	1.81E-01
	134	Phospholipid levels in medium HDL || id: ebi-a-GCST90092900	Body mass index || id: ieu-a-835	66	-1.09E-01	3.05E-02	3.63E-04	8.76E-04	-1.60E-04	2.45E-03	9.48E-01
	135	Phospholipids to total lipids ratio in medium HDL || id: ebi-a-GCST90092901	Body mass index || id: ieu-a-835	65	2.04E-01	4.17E-02	1.02E-06	1.01E-05	4.92E-03	3.27E-03	1.38E-01
	136	Phospholipids to total lipids ratio in medium LDL || id: ebi-a-GCST90092913	Body mass index || id: ieu-a-835	68	5.74E-02	2.37E-02	1.52E-02	2.27E-02	2.21E-03	1.89E-03	2.47E-01
	137	Phospholipids to total lipids ratio in medium VLDL || id: ebi-a-GCST90092925	Body mass index || id: ieu-a-835	67	-9.61E-02	3.15E-02	2.29E-03	4.11E-03	-2.31E-03	2.51E-03	3.62E-01
	138	Phospholipids to total lipids ratio in small VLDL || id: ebi-a-GCST90092977	Body mass index || id: ieu-a-835	66	-1.39E-01	3.18E-02	1.19E-05	5.39E-05	-1.80E-03	2.52E-03	4.79E-01
	139	Total phospholipid levels in lipoprotein particles || id: ebi-a-GCST90092991	Body mass index || id: ieu-a-835	65	-9.36E-02	3.04E-02	2.06E-03	3.77E-03	2.24E-04	2.42E-03	9.26E-01
	140	Phospholipid levels in VLDL || id: ebi-a-GCST90093001	Body mass index || id: ieu-a-835	68	6.56E-02	2.74E-02	1.69E-02	2.49E-02	1.57E-03	2.21E-03	4.80E-01
	141	Phospholipid levels in very large HDL || id: ebi-a-GCST90093012	Body mass index || id: ieu-a-835	65	-1.81E-01	3.82E-02	2.05E-06	1.50E-05	-4.41E-03	2.99E-03	1.46E-01
	142	Phospholipids to total lipids ratio in very large HDL || id: ebi-a-GCST90093013	Body mass index || id: ieu-a-835	67	-1.60E-01	3.35E-02	1.65E-06	1.33E-05	-4.06E-03	2.66E-03	1.32E-01
	143	Phospholipid levels in very large VLDL || id: ebi-a-GCST90093024	Body mass index || id: ieu-a-835	68	1.17E-01	3.03E-02	1.12E-04	3.36E-04	2.28E-03	2.43E-03	3.52E-01
	144	Phospholipids to total lipids ratio in very small VLDL || id: ebi-a-GCST90093037	Body mass index || id: ieu-a-835	66	1.86E-01	3.16E-02	4.11E-09	8.52E-08	3.11E-03	2.51E-03	2.19E-01
	145	Phospholipid levels in chylomicrons and extremely large VLDL || id: ebi-a-GCST90093048	Body mass index || id: ieu-a-835	68	1.28E-01	3.10E-02	3.60E-05	1.41E-04	2.91E-03	2.48E-03	2.45E-01
	146	Phospholipids to total lipids ratio in chylomicrons and extremely large VLDL || id: ebi-a-GCST90093049	Body mass index || id: ieu-a-835	68	1.07E-01	3.06E-02	4.79E-04	1.12E-03	1.22E-03	2.47E-03	6.24E-01
Total lipid levels	147	Total lipid levels in HDL || id: ebi-a-GCST90092825	Body mass index || id: ieu-a-835	66	-1.71E-01	3.62E-02	2.20E-06	1.52E-05	-2.20E-03	2.90E-03	4.50E-01
	148	Total lipid levels in IDL || id: ebi-a-GCST90092837	Body mass index || id: ieu-a-835	67	-9.99E-02	3.25E-02	2.14E-03	3.90E-03	1.02E-03	2.61E-03	6.98E-01
	149	Total lipid levels in large HDL || id: ebi-a-GCST90092850	Body mass index || id: ieu-a-835	64	-2.12E-01	3.54E-02	2.04E-09	6.69E-08	-1.92E-03	2.82E-03	4.97E-01
	150	Total lipid levels in large LDL || id: ebi-a-GCST90092862	Body mass index || id: ieu-a-835	67	-7.75E-02	3.17E-02	1.45E-02	2.17E-02	4.48E-04	2.54E-03	8.61E-01
	151	Total lipid levels in large VLDL || id: ebi-a-GCST90092874	Body mass index || id: ieu-a-835	68	9.93E-02	2.93E-02	7.11E-04	1.54E-03	2.36E-03	2.35E-03	3.20E-01
	152	Total lipid levels in medium HDL || id: ebi-a-GCST90092898	Body mass index || id: ieu-a-835	66	-1.30E-01	3.23E-02	5.31E-05	1.91E-04	-7.30E-04	2.59E-03	7.79E-01
	153	Total lipid levels in lipoprotein particles || id: ebi-a-GCST90092989	Body mass index || id: ieu-a-835	67	-7.38E-02	3.07E-02	1.63E-02	2.42E-02	1.01E-03	2.46E-03	6.84E-01
	154	Total lipid levels in VLDL || id: ebi-a-GCST90092999	Body mass index || id: ieu-a-835	68	7.82E-02	2.77E-02	4.74E-03	7.87E-03	1.83E-03	2.23E-03	4.15E-01
	155	Total lipid levels in very large HDL || id: ebi-a-GCST90093010	Body mass index || id: ieu-a-835	65	-1.88E-01	3.93E-02	1.83E-06	1.42E-05	-4.51E-03	3.08E-03	1.49E-01
	156	Total lipid levels in very large VLDL || id: ebi-a-GCST90093022	Body mass index || id: ieu-a-835	68	1.19E-01	3.06E-02	9.80E-05	3.05E-04	2.50E-03	2.46E-03	3.13E-01
	157	Total lipid levels in chylomicrons and extremely large VLDL || id: ebi-a-GCST90093046	Body mass index || id: ieu-a-835	68	1.18E-01	3.03E-02	9.52E-05	3.00E-04	2.56E-03	2.43E-03	2.96E-01
Fatty acid levels	158	Ratio of docosahexaenoic acid to total fatty acid levels || id: ebi-a-GCST90092817	Body mass index || id: ieu-a-835	68	-1.10E-01	2.66E-02	3.62E-05	1.41E-04	1.04E-04	2.15E-03	9.61E-01
	159	Ratio of linoleic acid to total fatty acids || id: ebi-a-GCST90092881	Body mass index || id: ieu-a-835	67	-1.66E-01	3.04E-02	4.67E-08	6.46E-07	-4.12E-03	2.38E-03	8.87E-02
	160	Monounsaturated fatty acid levels || id: ebi-a-GCST90092928	Body mass index || id: ieu-a-835	68	7.86E-02	2.61E-02	2.60E-03	4.53E-03	2.61E-03	2.09E-03	2.16E-01
	161	Ratio of monounsaturated fatty acids to total fatty acids || id: ebi-a-GCST90092929	Body mass index || id: ieu-a-835	67	1.87E-01	3.06E-02	1.01E-09	5.03E-08	4.39E-03	2.42E-03	7.36E-02
	162	Omega-6 fatty acid levels || id: ebi-a-GCST90092933	Body mass index || id: ieu-a-835	66	-6.60E-02	2.80E-02	1.84E-02	2.68E-02	1.17E-03	2.23E-03	6.02E-01
	163	Ratio of omega-6 fatty acids to total fatty acids || id: ebi-a-GCST90092935	Body mass index || id: ieu-a-835	68	-1.44E-01	2.95E-02	1.09E-06	1.01E-05	-3.48E-03	2.35E-03	1.43E-01
	164	Polyunsaturated fatty acid levels || id: ebi-a-GCST90092939	Body mass index || id: ieu-a-835	67	-7.33E-02	2.99E-02	1.42E-02	2.16E-02	1.22E-03	2.40E-03	6.14E-01
	165	Ratio of polyunsaturated fatty acids to monounsaturated fatty acids || id: ebi-a-GCST90092940	Body mass index || id: ieu-a-835	68	-1.79E-01	3.15E-02	1.23E-08	1.91E-07	-3.34E-03	2.51E-03	1.89E-01
	166	Ratio of polyunsaturated fatty acids to total fatty acids || id: ebi-a-GCST90092941	Body mass index || id: ieu-a-835	68	-1.44E-01	2.95E-02	1.08E-06	1.01E-05	-3.17E-03	2.35E-03	1.82E-01
	167	Docosahexaenoic acid levels || id: ebi-a-GCST90092816	Body mass index || id: ieu-a-835	68	-1.13E-01	2.91E-02	1.10E-04	3.34E-04	1.29E-03	2.35E-03	5.84E-01
	168	Linoleic acid levels || id: ebi-a-GCST90092880	Body mass index || id: ieu-a-835	66	-7.86E-02	2.64E-02	2.91E-03	4.99E-03	-1.70E-05	2.10E-03	9.94E-01
	169	Degree of unsaturation || id: ebi-a-GCST90092994	Body mass index || id: ieu-a-835	68	-1.16E-01	2.92E-02	7.23E-05	2.43E-04	-5.31E-04	2.36E-03	8.23E-01
Other metabolites	170	Acetoacetate levels || id: ebi-a-GCST90092804	Body mass index || id: ieu-a-835	68	9.26E-02	2.65E-02	4.86E-04	1.12E-03	2.96E-04	2.15E-03	8.91E-01
	171	Albumin levels || id: ebi-a-GCST90092807	Body mass index || id: ieu-a-835	68	-1.07E-01	2.79E-02	1.34E-04	3.78E-04	1.19E-03	2.25E-03	5.99E-01
	172	Apolipoprotein A1 levels || id: ebi-a-GCST90092808	Body mass index || id: ieu-a-835	66	-1.61E-01	3.48E-02	3.86E-06	2.29E-05	-1.68E-03	2.79E-03	5.49E-01
	173	Ratio of apolipoprotein B to apolipoprotein A1 levels || id: ebi-a-GCST90092810	Body mass index || id: ieu-a-835	68	8.07E-02	3.22E-02	1.21E-02	1.88E-02	1.31E-04	2.60E-03	9.60E-01
	174	Glucose levels || id: ebi-a-GCST90092819	Body mass index || id: ieu-a-835	67	9.40E-02	2.48E-02	1.53E-04	4.21E-04	-1.74E-03	1.99E-03	3.86E-01
	175	Glycoprotein acetyls levels || id: ebi-a-GCST90092821	Body mass index || id: ieu-a-835	68	1.66E-01	2.91E-02	1.11E-08	1.85E-07	3.49E-03	2.31E-03	1.37E-01
	176	3-Hydroxybutyrate levels || id: ebi-a-GCST90092811	Body mass index || id: ieu-a-835	68	6.48E-02	2.98E-02	3.00E-02	4.26E-02	-8.03E-04	2.41E-03	7.40E-01

We identified metabolic traits associated with body mass index, filtered them based on the q-values obtained from the IVW method, and excluded those metabolic traits exhibiting horizontal pleiotropy. The IVW method, known for its heightened sensitivity in detecting causality, serves as our primary tool to ascertain the presence of a causal relationship. The MR-Egger method is also employed for detecting horizontal pleiotropy due to its allowance for the presence of non-zero intercepts (The columns in the table corresponding to “egger_intercept”, “se”, and “pval”), a p_value below 0.05 suggests the existence of horizontal pleiotropy. IVW: Inverse Variance Weighted method; MR_Egger: MR-Egger method; SNPs: Single-nucleotide polymorphisms; q_value: The P value post FDR method (Benjamini and Hochberg) corrected; pval: p-value.

**Table 3:** The metabolic traits associated with cholelithiasis in the FinnGen Biobank, filtered based on the q-values obtained from the IVW method, with the exclusion of metabolic traits exhibiting horizontal pleiotropy.

Category	Serial number	Outcome	Exposure	Number of SNPs	IVW_Beta	IVW_se	IVW_pval	IVW_q_value	IVW_OR	IVW_lci95	IVW_uci95	egger_intercept	se	pval
Amino acids	1	Cholelithiasis in the FinnGen Biobank	Alanine levels || id: ebi-a-GCST90092806	27	1.99E-01	6.72E-02	3.09E-03	1.51E-02	1.22E + 00	1.07E + 00	1.39E + 00	4.88E-03	7.65E-03	5.29E-01
Cholesteryl ester	2	Cholelithiasis in the FinnGen Biobank	Cholesteryl ester levels in IDL || id: ebi-a-GCST90092833	50	-1.06E-01	3.96E-02	7.67E-03	2.73E-02	9.00E-01	8.32E-01	9.72E-01	2.43E-03	3.98E-03	5.45E-01
	3	Cholelithiasis in the FinnGen Biobank	Cholesteryl ester levels in large HDL || id: ebi-a-GCST90092846	81	-8.21E-02	3.37E-02	1.48E-02	4.09E-02	9.21E-01	8.62E-01	9.84E-01	-4.06E-03	2.68E-03	1.33E-01
	4	Cholelithiasis in the FinnGen Biobank	Cholesteryl ester levels in large LDL || id: ebi-a-GCST90092858	37	-1.23E-01	4.61E-02	7.84E-03	2.75E-02	8.85E-01	8.08E-01	9.68E-01	2.77E-03	4.83E-03	5.70E-01
	5	Cholelithiasis in the FinnGen Biobank	Cholesteryl esters to total lipids ratio in large VLDL || id: ebi-a-GCST90092871	48	-1.12E-01	4.59E-02	1.49E-02	4.09E-02	8.94E-01	8.17E-01	9.78E-01	3.02E-03	4.53E-03	5.09E-01
	6	Cholelithiasis in the FinnGen Biobank	Cholesteryl ester levels in LDL || id: ebi-a-GCST90092884	33	-1.09E-01	4.24E-02	1.03E-02	3.29E-02	8.97E-01	8.25E-01	9.75E-01	6.54E-03	4.43E-03	1.50E-01
	7	Cholelithiasis in the FinnGen Biobank	Cholesteryl ester levels in medium LDL || id: ebi-a-GCST90092906	38	-1.42E-01	3.81E-02	1.89E-04	2.63E-03	8.67E-01	8.05E-01	9.35E-01	5.40E-03	3.75E-03	1.59E-01
	8	Cholelithiasis in the FinnGen Biobank	Cholesteryl ester levels in medium VLDL || id: ebi-a-GCST90092918	36	-1.53E-01	3.79E-02	5.49E-05	1.58E-03	8.58E-01	7.97E-01	9.24E-01	-1.76E-03	4.00E-03	6.63E-01
	9	Cholelithiasis in the FinnGen Biobank	Cholesteryl esters to total lipids ratio in medium VLDL || id: ebi-a-GCST90092919	54	-1.45E-01	4.88E-02	2.95E-03	1.51E-02	8.65E-01	7.86E-01	9.52E-01	2.50E-03	3.94E-03	5.29E-01
	10	Cholelithiasis in the FinnGen Biobank	Cholesteryl ester levels in small LDL || id: ebi-a-GCST90092958	39	-1.42E-01	3.60E-02	8.06E-05	1.58E-03	8.68E-01	8.08E-01	9.31E-01	3.82E-03	3.60E-03	2.95E-01
	11	Cholelithiasis in the FinnGen Biobank	Cholesteryl esters to total lipids ratio in small VLDL || id: ebi-a-GCST90092971	47	-1.06E-01	4.20E-02	1.16E-02	3.41E-02	8.99E-01	8.28E-01	9.77E-01	2.86E-03	4.14E-03	4.94E-01
	12	Cholelithiasis in the FinnGen Biobank	Cholesteryl ester levels in VLDL || id: ebi-a-GCST90092997	40	-1.70E-01	4.28E-02	7.11E-05	1.58E-03	8.44E-01	7.76E-01	9.18E-01	4.69E-03	4.09E-03	2.58E-01
	13	Cholelithiasis in the FinnGen Biobank	Cholesteryl ester levels in very large HDL || id: ebi-a-GCST90093006	72	-1.04E-01	4.32E-02	1.58E-02	4.19E-02	9.01E-01	8.28E-01	9.81E-01	-4.02E-03	3.62E-03	2.72E-01
	14	Cholelithiasis in the FinnGen Biobank	Cholesteryl esters to total lipids ratio in very large VLDL || id: ebi-a-GCST90093019	57	-1.54E-01	4.97E-02	1.99E-03	1.18E-02	8.58E-01	7.78E-01	9.45E-01	-7.45E-03	4.25E-03	8.49E-02
	15	Cholelithiasis in the FinnGen Biobank	Cholesteryl ester levels in very small VLDL || id: ebi-a-GCST90093030	43	-1.14E-01	4.08E-02	5.18E-03	2.08E-02	8.92E-01	8.24E-01	9.66E-01	4.47E-03	4.15E-03	2.88E-01
	16	Cholelithiasis in the FinnGen Biobank	Cholesteryl esters to total lipids ratio in chylomicrons and extremely large VLDL || id: ebi-a-GCST90093043	21	-1.38E-01	5.47E-02	1.18E-02	3.43E-02	8.71E-01	7.83E-01	9.70E-01	-8.42E-03	7.11E-03	2.51E-01
Free cholesterol	17	Cholelithiasis in the FinnGen Biobank	Free cholesterol levels in IDL || id: ebi-a-GCST90092835	46	-1.19E-01	4.40E-02	6.88E-03	2.56E-02	8.88E-01	8.15E-01	9.68E-01	-2.58E-04	4.53E-03	9.55E-01
	18	Cholelithiasis in the FinnGen Biobank	Free cholesterol to total lipids ratio in large HDL || id: ebi-a-GCST90092849	51	-1.04E-01	3.78E-02	5.75E-03	2.23E-02	9.01E-01	8.36E-01	9.70E-01	4.40E-03	3.64E-03	2.32E-01
	19	Cholelithiasis in the FinnGen Biobank	Free cholesterol levels in large LDL || id: ebi-a-GCST90092860	47	-1.14E-01	3.82E-02	2.90E-03	1.51E-02	8.93E-01	8.28E-01	9.62E-01	9.66E-04	3.70E-03	7.95E-01
	20	Cholelithiasis in the FinnGen Biobank	Free cholesterol levels in LDL || id: ebi-a-GCST90092885	38	-1.06E-01	3.83E-02	5.81E-03	2.23E-02	9.00E-01	8.35E-01	9.70E-01	3.76E-03	4.26E-03	3.83E-01
	21	Cholelithiasis in the FinnGen Biobank	Free cholesterol to total lipids ratio in medium HDL || id: ebi-a-GCST90092897	66	-8.13E-02	3.29E-02	1.35E-02	3.83E-02	9.22E-01	8.64E-01	9.83E-01	-1.09E-03	3.02E-03	7.18E-01
	22	Cholelithiasis in the FinnGen Biobank	Free cholesterol levels in medium LDL || id: ebi-a-GCST90092908	38	-1.01E-01	4.26E-02	1.83E-02	4.76E-02	9.04E-01	8.32E-01	9.83E-01	4.22E-03	4.42E-03	3.46E-01
	23	Cholelithiasis in the FinnGen Biobank	Free cholesterol levels in medium VLDL || id: ebi-a-GCST90092920	46	-1.65E-01	3.72E-02	8.82E-06	7.46E-04	8.48E-01	7.88E-01	9.12E-01	1.93E-03	3.43E-03	5.77E-01
	24	Cholelithiasis in the FinnGen Biobank	Free cholesterol to total lipids ratio in medium VLDL || id: ebi-a-GCST90092921	48	-1.63E-01	4.15E-02	8.88E-05	1.58E-03	8.50E-01	7.83E-01	9.22E-01	2.08E-03	3.75E-03	5.81E-01
	25	Cholelithiasis in the FinnGen Biobank	Free cholesterol to total lipids ratio in small HDL || id: ebi-a-GCST90092949	57	-8.93E-02	3.10E-02	3.95E-03	1.78E-02	9.15E-01	8.61E-01	9.72E-01	4.94E-03	3.76E-03	1.94E-01
	26	Cholelithiasis in the FinnGen Biobank	Free cholesterol levels in small LDL || id: ebi-a-GCST90092960	42	-1.08E-01	4.04E-02	7.61E-03	2.73E-02	8.98E-01	8.30E-01	9.72E-01	-6.42E-04	4.33E-03	8.83E-01
	27	Cholelithiasis in the FinnGen Biobank	Free cholesterol levels in small VLDL || id: ebi-a-GCST90092972	43	-1.65E-01	3.76E-02	1.20E-05	7.46E-04	8.48E-01	7.88E-01	9.13E-01	2.46E-03	3.81E-03	5.21E-01
	28	Cholelithiasis in the FinnGen Biobank	Total free cholesterol levels || id: ebi-a-GCST90092988	44	-1.54E-01	3.83E-02	6.22E-05	1.58E-03	8.58E-01	7.96E-01	9.25E-01	3.06E-03	3.58E-03	3.98E-01
	29	Cholelithiasis in the FinnGen Biobank	Free cholesterol levels in very large HDL || id: ebi-a-GCST90093008	55	-1.09E-01	4.27E-02	1.03E-02	3.29E-02	8.96E-01	8.24E-01	9.74E-01	2.29E-03	3.94E-03	5.64E-01
	30	Cholelithiasis in the FinnGen Biobank	Free cholesterol levels in very small VLDL || id: ebi-a-GCST90093032	41	-1.26E-01	3.81E-02	9.32E-04	8.38E-03	8.81E-01	8.18E-01	9.50E-01	7.75E-03	4.02E-03	6.08E-02
Cholesterol levels	31	Cholelithiasis in the FinnGen Biobank	Clinical LDL cholesterol levels || id: ebi-a-GCST90092814	34	-1.12E-01	4.20E-02	7.64E-03	2.73E-02	8.94E-01	8.23E-01	9.71E-01	1.56E-03	4.55E-03	7.34E-01
	32	Cholelithiasis in the FinnGen Biobank	Cholesterol levels in IDL || id: ebi-a-GCST90092831	49	-1.05E-01	4.01E-02	8.93E-03	3.01E-02	9.00E-01	8.32E-01	9.74E-01	2.60E-03	4.02E-03	5.20E-01
	33	Cholelithiasis in the FinnGen Biobank	Cholesterol levels in large HDL || id: ebi-a-GCST90092844	80	-9.18E-02	3.59E-02	1.06E-02	3.35E-02	9.12E-01	8.50E-01	9.79E-01	-5.10E-03	2.86E-03	7.80E-02
	34	Cholelithiasis in the FinnGen Biobank	Cholesterol to total lipids ratio in large HDL || id: ebi-a-GCST90092845	71	-1.04E-01	4.37E-02	1.73E-02	4.55E-02	9.01E-01	8.27E-01	9.82E-01	-4.78E-03	3.34E-03	1.57E-01
	35	Cholelithiasis in the FinnGen Biobank	Cholesterol levels in large LDL || id: ebi-a-GCST90092856	36	-1.23E-01	4.30E-02	4.08E-03	1.78E-02	8.84E-01	8.13E-01	9.62E-01	8.00E-04	4.52E-03	8.61E-01
	36	Cholelithiasis in the FinnGen Biobank	Cholesterol to total lipids ratio in large VLDL || id: ebi-a-GCST90092869	40	-1.37E-01	4.17E-02	9.82E-04	8.38E-03	8.72E-01	8.03E-01	9.46E-01	7.96E-04	4.91E-03	8.72E-01
	37	Cholelithiasis in the FinnGen Biobank	LDL cholesterol levels || id: ebi-a-GCST90092883	35	-1.10E-01	4.34E-02	1.10E-02	3.36E-02	8.95E-01	8.22E-01	9.75E-01	5.45E-03	4.50E-03	2.34E-01
	38	Cholelithiasis in the FinnGen Biobank	Cholesterol levels in medium LDL || id: ebi-a-GCST90092904	33	-1.31E-01	4.02E-02	1.15E-03	8.64E-03	8.78E-01	8.11E-01	9.49E-01	4.20E-03	4.19E-03	3.24E-01
	39	Cholelithiasis in the FinnGen Biobank	Cholesterol levels in medium VLDL || id: ebi-a-GCST90092916	35	-1.67E-01	3.81E-02	1.18E-05	7.46E-04	8.46E-01	7.85E-01	9.12E-01	-2.13E-03	3.91E-03	5.88E-01
	40	Cholelithiasis in the FinnGen Biobank	Cholesterol to total lipids ratio in medium VLDL || id: ebi-a-GCST90092917	56	-1.60E-01	4.87E-02	1.01E-03	8.38E-03	8.52E-01	7.74E-01	9.37E-01	-8.30E-05	3.88E-03	9.83E-01
	41	Cholelithiasis in the FinnGen Biobank	Total cholesterol minus HDL-C levels || id: ebi-a-GCST90092930	35	-1.48E-01	3.37E-02	1.13E-05	7.46E-04	8.62E-01	8.07E-01	9.21E-01	6.99E-04	3.52E-03	8.44E-01
	42	Cholelithiasis in the FinnGen Biobank	Remnant cholesterol (non-HDL, non-LDL -cholesterol) || id: ebi-a-GCST90092943	35	-1.51E-01	3.63E-02	3.11E-05	1.47E-03	8.60E-01	8.01E-01	9.23E-01	5.10E-03	3.57E-03	1.62E-01
	43	Cholelithiasis in the FinnGen Biobank	Cholesterol levels in small LDL || id: ebi-a-GCST90092956	36	-1.16E-01	3.31E-02	4.35E-04	4.52E-03	8.90E-01	8.34E-01	9.50E-01	5.88E-03	3.35E-03	8.87E-02
	44	Cholelithiasis in the FinnGen Biobank	Cholesterol levels in small VLDL || id: ebi-a-GCST90092968	46	-1.58E-01	4.39E-02	3.14E-04	3.72E-03	8.54E-01	7.83E-01	9.30E-01	5.62E-03	4.15E-03	1.83E-01
	45	Cholelithiasis in the FinnGen Biobank	Total esterified cholesterol levels || id: ebi-a-GCST90092986	46	-1.14E-01	4.02E-02	4.65E-03	1.96E-02	8.92E-01	8.25E-01	9.66E-01	7.23E-03	3.78E-03	6.25E-02
	46	Cholelithiasis in the FinnGen Biobank	Cholesterol to total lipids ratio in very large VLDL || id: ebi-a-GCST90093017	57	-1.64E-01	5.21E-02	1.61E-03	1.11E-02	8.48E-01	7.66E-01	9.40E-01	-7.14E-03	4.62E-03	1.28E-01
	47	Cholelithiasis in the FinnGen Biobank	Cholesterol levels in very small VLDL || id: ebi-a-GCST90093028	44	-1.40E-01	3.77E-02	2.06E-04	2.70E-03	8.69E-01	8.08E-01	9.36E-01	3.72E-03	3.81E-03	3.34E-01
Particle concentrations	48	Cholelithiasis in the FinnGen Biobank	Concentration of IDL particles || id: ebi-a-GCST90092838	36	-1.27E-01	4.09E-02	1.84E-03	1.17E-02	8.80E-01	8.13E-01	9.54E-01	2.87E-03	4.49E-03	5.26E-01
	49	Cholelithiasis in the FinnGen Biobank	Concentration of large HDL particles || id: ebi-a-GCST90092851	73	-8.10E-02	3.19E-02	1.11E-02	3.36E-02	9.22E-01	8.66E-01	9.82E-01	-3.43E-03	2.65E-03	2.00E-01
	50	Cholelithiasis in the FinnGen Biobank	Concentration of large LDL particles || id: ebi-a-GCST90092863	38	-1.30E-01	4.38E-02	2.91E-03	1.51E-02	8.78E-01	8.05E-01	9.56E-01	-5.08E-04	4.49E-03	9.11E-01
	51	Cholelithiasis in the FinnGen Biobank	Concentration of LDL particles || id: ebi-a-GCST90092887	34	-1.46E-01	3.90E-02	1.80E-04	2.63E-03	8.64E-01	8.00E-01	9.33E-01	3.96E-04	4.13E-03	9.24E-01
	52	Cholelithiasis in the FinnGen Biobank	Concentration of medium LDL particles || id: ebi-a-GCST90092911	35	-1.60E-01	3.87E-02	3.54E-05	1.47E-03	8.52E-01	7.90E-01	9.19E-01	2.24E-03	3.82E-03	5.61E-01
	53	Cholelithiasis in the FinnGen Biobank	Concentration of small LDL particles || id: ebi-a-GCST90092963	39	-1.48E-01	3.72E-02	7.28E-05	1.58E-03	8.63E-01	8.02E-01	9.28E-01	3.18E-03	3.76E-03	4.03E-01
Triglyceride levels	54	Cholelithiasis in the FinnGen Biobank	Triglyceride levels in IDL || id: ebi-a-GCST90092841	51	-8.75E-02	3.38E-02	9.61E-03	3.19E-02	9.16E-01	8.58E-01	9.79E-01	3.84E-03	3.25E-03	2.44E-01
	55	Cholelithiasis in the FinnGen Biobank	Triglyceride levels in large LDL || id: ebi-a-GCST90092866	49	-1.14E-01	3.65E-02	1.81E-03	1.17E-02	8.92E-01	8.31E-01	9.59E-01	1.48E-03	3.33E-03	6.59E-01
	56	Cholelithiasis in the FinnGen Biobank	Triglycerides to total lipids ratio in large VLDL || id: ebi-a-GCST90092879	39	1.04E-01	3.09E-02	8.00E-04	7.66E-03	1.11E + 00	1.04E + 00	1.18E + 00	1.36E-03	3.76E-03	7.20E-01
	57	Cholelithiasis in the FinnGen Biobank	Triglycerides to total lipids ratio in medium VLDL || id: ebi-a-GCST90092927	60	1.71E-01	4.37E-02	8.80E-05	1.58E-03	1.19E + 00	1.09E + 00	1.29E + 00	1.22E-03	3.49E-03	7.27E-01
	58	Cholelithiasis in the FinnGen Biobank	Triglycerides to total lipids ratio in small VLDL || id: ebi-a-GCST90092979	49	1.11E-01	4.21E-02	8.46E-03	2.88E-02	1.12E + 00	1.03E + 00	1.21E + 00	-6.46E-03	3.86E-03	1.01E-01
	59	Cholelithiasis in the FinnGen Biobank	Triglycerides to total lipids ratio in very large VLDL || id: ebi-a-GCST90093027	42	1.33E-01	5.26E-02	1.17E-02	3.41E-02	1.14E + 00	1.03E + 00	1.27E + 00	-2.05E-03	5.39E-03	7.06E-01
Phospholipid levels	60	Cholelithiasis in the FinnGen Biobank	Phospholipid levels in IDL || id: ebi-a-GCST90092839	45	-1.12E-01	3.86E-02	3.77E-03	1.74E-02	8.94E-01	8.29E-01	9.65E-01	5.01E-03	3.80E-03	1.94E-01
	61	Cholelithiasis in the FinnGen Biobank	Phospholipids to total lipids ratio in large HDL || id: ebi-a-GCST90092853	61	1.33E-01	4.64E-02	4.05E-03	1.78E-02	1.14E + 00	1.04E + 00	1.25E + 00	5.35E-04	4.00E-03	8.94E-01
	62	Cholelithiasis in the FinnGen Biobank	Phospholipid levels in large LDL || id: ebi-a-GCST90092864	36	-1.14E-01	4.28E-02	7.94E-03	2.75E-02	8.93E-01	8.21E-01	9.71E-01	2.43E-03	4.39E-03	5.83E-01
	63	Cholelithiasis in the FinnGen Biobank	Phospholipids to total lipids ratio in large LDL || id: ebi-a-GCST90092865	32	1.23E-01	4.18E-02	3.30E-03	1.58E-02	1.13E + 00	1.04E + 00	1.23E + 00	5.61E-03	5.21E-03	2.90E-01
	64	Cholelithiasis in the FinnGen Biobank	Phospholipid levels in LDL || id: ebi-a-GCST90092888	38	-1.07E-01	4.42E-02	1.58E-02	4.19E-02	8.99E-01	8.24E-01	9.80E-01	3.00E-03	4.50E-03	5.10E-01
	65	Cholelithiasis in the FinnGen Biobank	Phospholipid levels in medium LDL || id: ebi-a-GCST90092912	36	-9.93E-02	4.09E-02	1.52E-02	4.12E-02	9.05E-01	8.36E-01	9.81E-01	6.92E-03	4.42E-03	1.27E-01
	66	Cholelithiasis in the FinnGen Biobank	Phospholipid levels in medium VLDL || id: ebi-a-GCST90092924	49	-1.52E-01	4.30E-02	4.02E-04	4.35E-03	8.59E-01	7.89E-01	9.34E-01	6.84E-03	3.71E-03	7.18E-02
	67	Cholelithiasis in the FinnGen Biobank	Phospholipids to total lipids ratio in medium VLDL || id: ebi-a-GCST90092925	44	-1.27E-01	4.11E-02	1.93E-03	1.18E-02	8.80E-01	8.12E-01	9.54E-01	3.18E-03	4.27E-03	4.61E-01
	68	Cholelithiasis in the FinnGen Biobank	Phospholipid levels in small LDL || id: ebi-a-GCST90092964	38	-1.04E-01	3.74E-02	5.25E-03	2.08E-02	9.01E-01	8.37E-01	9.69E-01	1.04E-03	4.17E-03	8.05E-01
	69	Cholelithiasis in the FinnGen Biobank	Phospholipids to total lipids ratio in small VLDL || id: ebi-a-GCST90092977	53	-1.13E-01	4.13E-02	6.45E-03	2.43E-02	8.94E-01	8.24E-01	9.69E-01	4.05E-03	3.62E-03	2.68E-01
	70	Cholelithiasis in the FinnGen Biobank	Phospholipid levels in VLDL || id: ebi-a-GCST90093001	37	-1.19E-01	4.84E-02	1.37E-02	3.84E-02	8.88E-01	8.07E-01	9.76E-01	4.62E-04	5.20E-03	9.30E-01
	71	Cholelithiasis in the FinnGen Biobank	Phospholipids to total lipids ratio in very large VLDL || id: ebi-a-GCST90093025	30	-1.52E-01	6.06E-02	1.22E-02	3.50E-02	8.59E-01	7.63E-01	9.67E-01	8.01E-03	5.88E-03	1.84E-01
	72	Cholelithiasis in the FinnGen Biobank	Phospholipid levels in very small VLDL || id: ebi-a-GCST90093036	47	-1.17E-01	3.64E-02	1.26E-03	9.22E-03	8.89E-01	8.28E-01	9.55E-01	5.14E-03	3.65E-03	1.66E-01
	73	Cholelithiasis in the FinnGen Biobank	Sphingomyelin levels || id: ebi-a-GCST90092982	51	-1.21E-01	3.72E-02	1.11E-03	8.64E-03	8.86E-01	8.24E-01	9.53E-01	5.22E-03	3.68E-03	1.63E-01
Total lipid	74	Cholelithiasis in the FinnGen Biobank	Total lipid levels in IDL || id: ebi-a-GCST90092837	46	-1.06E-01	4.11E-02	1.01E-02	3.29E-02	9.00E-01	8.30E-01	9.75E-01	5.15E-03	4.24E-03	2.30E-01
	75	Cholelithiasis in the FinnGen Biobank	Total lipid levels in large LDL || id: ebi-a-GCST90092862	36	-1.27E-01	4.29E-02	3.08E-03	1.51E-02	8.81E-01	8.10E-01	9.58E-01	1.15E-03	4.60E-03	8.04E-01
	76	Cholelithiasis in the FinnGen Biobank	Total lipid levels in LDL || id: ebi-a-GCST90092886	36	-1.14E-01	4.07E-02	4.97E-03	2.06E-02	8.92E-01	8.23E-01	9.66E-01	3.87E-03	4.31E-03	3.75E-01
	77	Cholelithiasis in the FinnGen Biobank	Total lipid levels in medium LDL || id: ebi-a-GCST90092910	35	-1.25E-01	3.84E-02	1.12E-03	8.64E-03	8.82E-01	8.18E-01	9.51E-01	5.74E-03	4.01E-03	1.61E-01
	78	Cholelithiasis in the FinnGen Biobank	Total lipid levels in small LDL || id: ebi-a-GCST90092962	36	-1.21E-01	3.42E-02	3.92E-04	4.35E-03	8.86E-01	8.28E-01	9.47E-01	3.32E-03	3.70E-03	3.75E-01
	79	Cholelithiasis in the FinnGen Biobank	Total lipid levels in very small VLDL || id: ebi-a-GCST90093034	44	-1.18E-01	3.66E-02	1.33E-03	9.49E-03	8.89E-01	8.27E-01	9.55E-01	7.88E-03	3.93E-03	5.14E-02
Fatty acid	80	Cholelithiasis in the FinnGen Biobank	Ratio of docosahexaenoic acid to total fatty acid levels || id: ebi-a-GCST90092817	20	-2.56E-01	6.87E-02	1.90E-04	2.63E-03	7.74E-01	6.77E-01	8.85E-01	3.87E-03	7.25E-03	6.00E-01
	81	Cholelithiasis in the FinnGen Biobank	Omega-6 fatty acid levels || id: ebi-a-GCST90092933	41	-1.42E-01	4.85E-02	3.44E-03	1.62E-02	8.68E-01	7.89E-01	9.54E-01	6.49E-03	5.47E-03	2.43E-01
	82	Cholelithiasis in the FinnGen Biobank	Linoleic acid levels || id: ebi-a-GCST90092880	32	-1.62E-01	5.46E-02	2.98E-03	1.51E-02	8.50E-01	7.64E-01	9.46E-01	1.27E-02	6.62E-03	6.44E-02
	83	Cholelithiasis in the FinnGen Biobank	Degree of unsaturation || id: ebi-a-GCST90092994	27	-2.20E-01	6.68E-02	9.93E-04	8.38E-03	8.03E-01	7.04E-01	9.15E-01	-4.23E-03	6.58E-03	5.26E-01
Other metabolites	84	Cholelithiasis in the FinnGen Biobank	Apolipoprotein B levels || id: ebi-a-GCST90092809	35	-1.46E-01	3.99E-02	2.51E-04	3.12E-03	8.64E-01	7.99E-01	9.34E-01	8.82E-04	4.36E-03	8.41E-01
	85	Cholelithiasis in the FinnGen Biobank	Ratio of apolipoprotein B to apolipoprotein A1 levels || id: ebi-a-GCST90092810	53	-1.26E-01	3.07E-02	4.14E-05	1.47E-03	8.82E-01	8.30E-01	9.36E-01	4.85E-03	2.84E-03	9.39E-02

We identified metabolic traits associated with cholelithiasis in the FinnGen Biobank, filtered them based on the q-values obtained from the IVW method, and excluded those metabolic traits exhibiting horizontal pleiotropy. The IVW method, known for its heightened sensitivity in detecting causality, serves as our primary tool to ascertain the presence of a causal relationship. The MR-Egger method is also employed for detecting horizontal pleiotropy due to its allowance for the presence of non-zero intercepts (The columns in the table corresponding to “egger_intercept”, “se”, and “pval”), a p_value below 0.05 suggests the existence of horizontal pleiotropy. IVW: Inverse Variance Weighted method; MR_Egger: MR-Egger method; SNPs: Single-nucleotide polymorphisms; q_value: The P value post FDR method (Benjamini and Hochberg) corrected; pval: p-value; OR: Odds Ratio; lci95: Lower Confidence Interval 95%; uci95: Upper Confidence Interval 95%.

**Table 4:** Mediators between body mass index (ID: ieu-a-835) and cholelithiasis in the FinnGen Biobank were identified based on their q-values, excluding metabolic traits that exhibited horizontal pleiotropy.

Classification	Index	The impact of body mass index(GIANT consortium id: ieu-a-835) on potential mediators										The impact of potential mediators on cholelithiasis (Round 10 releases of the FinnGen Biobank)										The mediation effect	The proportion of the mediation effect to the total effect
		Outcome	Exposure	Number of SNPs	IVW_Beta	IVW_se	IVW_pval	IVW_q_value	egger_intercept	se	pval	Outcome	Exposure	Number of SNPs	IVW_Beta	IVW_se	IVW_pval	IVW_q_value	egger_intercept	se	pval		
Cholesteryl ester levels	1	Cholesteryl ester levels in IDL || id: ebi-a-GCST90092833	Body mass index || id: ieu-a-835	67	-1.14E-01	3.40E-02	7.85E-04	1.69E-03	1.44E-03	2.72E-03	5.99E-01	Cholelithiasis in the FinnGen Biobank	Cholesteryl ester levels in IDL || id: ebi-a-GCST90092833	50	-1.06E-01	3.96E-02	7.67E-03	2.73E-02	2.43E-03	3.98E-03	5.45E-01	1.21E-02	2.82%
	2	Cholesteryl ester levels in large HDL || id: ebi-a-GCST90092846	Body mass index || id: ieu-a-835	63	-2.22E-01	3.62E-02	9.53E-10	5.03E-08	-1.72E-03	2.88E-03	5.52E-01	Cholelithiasis in the FinnGen Biobank	Cholesteryl ester levels in large HDL || id: ebi-a-GCST90092846	81	-8.21E-02	3.37E-02	1.48E-02	4.09E-02	-4.06E-03	2.68E-03	1.33E-01	1.82E-02	4.25%
	3	Cholesteryl ester levels in large LDL || id: ebi-a-GCST90092858	Body mass index || id: ieu-a-835	67	-8.00E-02	3.20E-02	1.25E-02	1.92E-02	3.79E-04	2.57E-03	8.83E-01	Cholelithiasis in the FinnGen Biobank	Cholesteryl ester levels in large LDL || id: ebi-a-GCST90092858	37	-1.23E-01	4.61E-02	7.84E-03	2.75E-02	2.77E-03	4.83E-03	5.70E-01	9.80E-03	2.29%
	4	Cholesteryl esters to total lipids ratio in large VLDL || id: ebi-a-GCST90092871	Body mass index || id: ieu-a-835	67	-9.80E-02	3.01E-02	1.11E-03	2.19E-03	-3.11E-03	2.38E-03	1.96E-01	Cholelithiasis in the FinnGen Biobank	Cholesteryl esters to total lipids ratio in large VLDL || id: ebi-a-GCST90092871	48	-1.12E-01	4.59E-02	1.49E-02	4.09E-02	3.02E-03	4.53E-03	5.09E-01	1.10E-02	2.56%
	5	Cholesteryl ester levels in medium VLDL || id: ebi-a-GCST90092918	Body mass index || id: ieu-a-835	67	-7.56E-02	2.87E-02	8.33E-03	1.31E-02	3.57E-04	2.30E-03	8.77E-01	Cholelithiasis in the FinnGen Biobank	Cholesteryl ester levels in medium VLDL || id: ebi-a-GCST90092918	36	-1.53E-01	3.79E-02	5.49E-05	1.58E-03	-1.76E-03	4.00E-03	6.63E-01	1.16E-02	2.70%
	6	Cholesteryl esters to total lipids ratio in medium VLDL || id: ebi-a-GCST90092919	Body mass index || id: ieu-a-835	67	-1.31E-01	3.55E-02	2.38E-04	6.16E-04	-3.51E-03	2.82E-03	2.17E-01	Cholelithiasis in the FinnGen Biobank	Cholesteryl esters to total lipids ratio in medium VLDL || id: ebi-a-GCST90092919	54	-1.45E-01	4.88E-02	2.95E-03	1.51E-02	2.50E-03	3.94E-03	5.29E-01	1.89E-02	4.43%
	7	Cholesteryl ester levels in very large HDL || id: ebi-a-GCST90093006	Body mass index || id: ieu-a-835	64	-2.25E-01	3.79E-02	2.96E-09	6.69E-08	-2.79E-03	3.00E-03	3.57E-01	Cholelithiasis in the FinnGen Biobank	Cholesteryl ester levels in very large HDL || id: ebi-a-GCST90093006	72	-1.04E-01	4.32E-02	1.58E-02	4.19E-02	-4.02E-03	3.62E-03	2.72E-01	2.34E-02	5.48%
	8	Cholesteryl esters to total lipids ratio in very large VLDL || id: ebi-a-GCST90093019	Body mass index || id: ieu-a-835	68	-1.36E-01	3.36E-02	5.37E-05	1.91E-04	-3.89E-03	2.67E-03	1.50E-01	Cholelithiasis in the FinnGen Biobank	Cholesteryl esters to total lipids ratio in very large VLDL || id: ebi-a-GCST90093019	57	-1.54E-01	4.97E-02	1.99E-03	1.18E-02	-7.45E-03	4.25E-03	8.49E-02	2.09E-02	4.87%
	9	Cholesteryl ester levels in very small VLDL || id: ebi-a-GCST90093030	Body mass index || id: ieu-a-835	67	-8.76E-02	2.91E-02	2.57E-03	4.51E-03	7.65E-04	2.33E-03	7.44E-01	Cholelithiasis in the FinnGen Biobank	Cholesteryl ester levels in very small VLDL || id: ebi-a-GCST90093030	43	-1.14E-01	4.08E-02	5.18E-03	2.08E-02	4.47E-03	4.15E-03	2.88E-01	9.99E-03	2.34%
	10	Cholesteryl esters to total lipids ratio in chylomicrons and extremely large VLDL || id: ebi-a-GCST90093043	Body mass index || id: ieu-a-835	68	-8.70E-02	2.48E-02	4.51E-04	1.06E-03	-1.84E-03	1.99E-03	3.59E-01	Cholelithiasis in the FinnGen Biobank	Cholesteryl esters to total lipids ratio in chylomicrons and extremely large VLDL || id: ebi-a-GCST90093043	21	-1.38E-01	5.47E-02	1.18E-02	3.43E-02	-8.42E-03	7.11E-03	2.51E-01	1.20E-02	2.80%
Free cholesterol levels	11	Free cholesterol levels in IDL || id: ebi-a-GCST90092835	Body mass index || id: ieu-a-835	67	-1.04E-01	3.31E-02	1.66E-03	3.05E-03	2.15E-04	2.65E-03	9.36E-01	Cholelithiasis in the FinnGen Biobank	Free cholesterol levels in IDL || id: ebi-a-GCST90092835	46	-1.19E-01	4.40E-02	6.88E-03	2.56E-02	-2.58E-04	4.53E-03	9.55E-01	1.24E-02	2.89%
	12	Free cholesterol to total lipids ratio in large HDL || id: ebi-a-GCST90092849	Body mass index || id: ieu-a-835	67	-1.47E-01	3.87E-02	1.54E-04	4.21E-04	-3.01E-03	3.09E-03	3.33E-01	Cholelithiasis in the FinnGen Biobank	Free cholesterol to total lipids ratio in large HDL || id: ebi-a-GCST90092849	51	-1.04E-01	3.78E-02	5.75E-03	2.23E-02	4.40E-03	3.64E-03	2.32E-01	1.53E-02	3.58%
	13	Free cholesterol levels in large LDL || id: ebi-a-GCST90092860	Body mass index || id: ieu-a-835	67	-1.09E-01	3.39E-02	1.33E-03	2.57E-03	1.74E-04	2.72E-03	9.49E-01	Cholelithiasis in the FinnGen Biobank	Free cholesterol levels in large LDL || id: ebi-a-GCST90092860	47	-1.14E-01	3.82E-02	2.90E-03	1.51E-02	9.66E-04	3.70E-03	7.95E-01	1.24E-02	2.89%
	14	Free cholesterol levels in LDL || id: ebi-a-GCST90092885	Body mass index || id: ieu-a-835	67	-9.04E-02	3.29E-02	6.01E-03	9.84E-03	1.91E-04	2.64E-03	9.43E-01	Cholelithiasis in the FinnGen Biobank	Free cholesterol levels in LDL || id: ebi-a-GCST90092885	38	-1.06E-01	3.83E-02	5.81E-03	2.23E-02	3.76E-03	4.26E-03	3.83E-01	9.56E-03	2.23%
	15	Free cholesterol to total lipids ratio in medium HDL || id: ebi-a-GCST90092897	Body mass index || id: ieu-a-835	64	-1.65E-01	3.84E-02	1.77E-05	7.72E-05	-2.55E-03	3.04E-03	4.04E-01	Cholelithiasis in the FinnGen Biobank	Free cholesterol to total lipids ratio in medium HDL || id: ebi-a-GCST90092897	66	-8.13E-02	3.29E-02	1.35E-02	3.83E-02	-1.09E-03	3.02E-03	7.18E-01	1.34E-02	3.13%
	16	Free cholesterol to total lipids ratio in medium VLDL || id: ebi-a-GCST90092921	Body mass index || id: ieu-a-835	67	-1.14E-01	3.36E-02	6.84E-04	1.49E-03	-3.20E-03	2.67E-03	2.35E-01	Cholelithiasis in the FinnGen Biobank	Free cholesterol to total lipids ratio in medium VLDL || id: ebi-a-GCST90092921	48	-1.63E-01	4.15E-02	8.88E-05	1.58E-03	2.08E-03	3.75E-03	5.81E-01	1.86E-02	4.34%
	17	Free cholesterol to total lipids ratio in small HDL || id: ebi-a-GCST90092949	Body mass index || id: ieu-a-835	67	-1.50E-01	3.88E-02	1.15E-04	3.42E-04	-3.32E-03	3.09E-03	2.86E-01	Cholelithiasis in the FinnGen Biobank	Free cholesterol to total lipids ratio in small HDL || id: ebi-a-GCST90092949	57	-8.93E-02	3.10E-02	3.95E-03	1.78E-02	4.94E-03	3.76E-03	1.94E-01	1.34E-02	3.12%
	18	Total free cholesterol levels || id: ebi-a-GCST90092988	Body mass index || id: ieu-a-835	67	-1.03E-01	3.25E-02	1.49E-03	2.77E-03	5.79E-04	2.61E-03	8.25E-01	Cholelithiasis in the FinnGen Biobank	Total free cholesterol levels || id: ebi-a-GCST90092988	44	-1.54E-01	3.83E-02	6.22E-05	1.58E-03	3.06E-03	3.58E-03	3.98E-01	1.59E-02	3.71%
	19	Free cholesterol levels in very large HDL || id: ebi-a-GCST90093008	Body mass index || id: ieu-a-835	66	-1.70E-01	3.82E-02	8.32E-06	4.32E-05	-4.04E-03	3.03E-03	1.88E-01	Cholelithiasis in the FinnGen Biobank	Free cholesterol levels in very large HDL || id: ebi-a-GCST90093008	55	-1.09E-01	4.27E-02	1.03E-02	3.29E-02	2.29E-03	3.94E-03	5.64E-01	1.87E-02	4.36%
Cholesterol levels	20	Clinical LDL cholesterol levels || id: ebi-a-GCST90092814	Body mass index || id: ieu-a-835	67	-6.79E-02	3.05E-02	2.60E-02	3.72E-02	6.12E-04	2.45E-03	8.03E-01	Cholelithiasis in the FinnGen Biobank	Clinical LDL cholesterol levels || id: ebi-a-GCST90092814	34	-1.12E-01	4.20E-02	7.64E-03	2.73E-02	1.56E-03	4.55E-03	7.34E-01	7.61E-03	1.78%
	21	Cholesterol levels in IDL || id: ebi-a-GCST90092831	Body mass index || id: ieu-a-835	67	-1.12E-01	3.38E-02	9.20E-04	1.89E-03	1.13E-03	2.71E-03	6.78E-01	Cholelithiasis in the FinnGen Biobank	Cholesterol levels in IDL || id: ebi-a-GCST90092831	49	-1.05E-01	4.01E-02	8.93E-03	3.01E-02	2.60E-03	4.02E-03	5.20E-01	1.18E-02	2.75%
	22	Cholesterol levels in large HDL || id: ebi-a-GCST90092844	Body mass index || id: ieu-a-835	63	-2.19E-01	3.60E-02	1.24E-09	5.14E-08	-1.72E-03	2.86E-03	5.50E-01	Cholelithiasis in the FinnGen Biobank	Cholesterol levels in large HDL || id: ebi-a-GCST90092844	80	-9.18E-02	3.59E-02	1.06E-02	3.35E-02	-5.10E-03	2.86E-03	7.80E-02	2.01E-02	4.69%
	23	Cholesterol to total lipids ratio in large HDL || id: ebi-a-GCST90092845	Body mass index || id: ieu-a-835	65	-1.96E-01	4.14E-02	2.13E-06	1.52E-05	-5.22E-03	3.24E-03	1.12E-01	Cholelithiasis in the FinnGen Biobank	Cholesterol to total lipids ratio in large HDL || id: ebi-a-GCST90092845	71	-1.04E-01	4.37E-02	1.73E-02	4.55E-02	-4.78E-03	3.34E-03	1.57E-01	2.04E-02	4.77%
	24	Cholesterol levels in large LDL || id: ebi-a-GCST90092856	Body mass index || id: ieu-a-835	67	-8.86E-02	3.26E-02	6.57E-03	1.05E-02	3.17E-04	2.62E-03	9.04E-01	Cholelithiasis in the FinnGen Biobank	Cholesterol levels in large LDL || id: ebi-a-GCST90092856	36	-1.23E-01	4.30E-02	4.08E-03	1.78E-02	8.00E-04	4.52E-03	8.61E-01	1.09E-02	2.56%
	25	LDL cholesterol levels || id: ebi-a-GCST90092883	Body mass index || id: ieu-a-835	67	-6.55E-02	3.07E-02	3.28E-02	4.64E-02	4.06E-04	2.46E-03	8.70E-01	Cholelithiasis in the FinnGen Biobank	LDL cholesterol levels || id: ebi-a-GCST90092883	35	-1.10E-01	4.34E-02	1.10E-02	3.36E-02	5.45E-03	4.50E-03	2.34E-01	7.23E-03	1.69%
	26	Cholesterol to total lipids ratio in medium VLDL || id: ebi-a-GCST90092917	Body mass index || id: ieu-a-835	67	-1.27E-01	3.52E-02	3.16E-04	7.87E-04	-3.49E-03	2.79E-03	2.15E-01	Cholelithiasis in the FinnGen Biobank	Cholesterol to total lipids ratio in medium VLDL || id: ebi-a-GCST90092917	56	-1.60E-01	4.87E-02	1.01E-03	8.38E-03	-8.30E-05	3.88E-03	9.83E-01	2.03E-02	4.74%
	27	Total esterified cholesterol levels || id: ebi-a-GCST90092986	Body mass index || id: ieu-a-835	65	-1.08E-01	3.32E-02	1.16E-03	2.27E-03	-3.31E-04	2.64E-03	9.01E-01	Cholelithiasis in the FinnGen Biobank	Total esterified cholesterol levels || id: ebi-a-GCST90092986	46	-1.14E-01	4.02E-02	4.65E-03	1.96E-02	7.23E-03	3.78E-03	6.25E-02	1.23E-02	2.87%
	28	Cholesterol to total lipids ratio in very large VLDL || id: ebi-a-GCST90093017	Body mass index || id: ieu-a-835	67	-1.23E-01	3.20E-02	1.25E-04	3.63E-04	-3.86E-03	2.52E-03	1.30E-01	Cholelithiasis in the FinnGen Biobank	Cholesterol to total lipids ratio in very large VLDL || id: ebi-a-GCST90093017	57	-1.64E-01	5.21E-02	1.61E-03	1.11E-02	-7.14E-03	4.62E-03	1.28E-01	2.01E-02	4.71%
	29	Cholesterol levels in very small VLDL || id: ebi-a-GCST90093028	Body mass index || id: ieu-a-835	67	-6.97E-02	2.79E-02	1.24E-02	1.91E-02	7.51E-04	2.24E-03	7.38E-01	Cholelithiasis in the FinnGen Biobank	Cholesterol levels in very small VLDL || id: ebi-a-GCST90093028	44	-1.40E-01	3.77E-02	2.06E-04	2.70E-03	3.72E-03	3.81E-03	3.34E-01	9.76E-03	2.28%
Particle concentrations	30	Concentration of large HDL particles || id: ebi-a-GCST90092851	Body mass index || id: ieu-a-835	63	-2.17E-01	3.52E-02	7.11E-10	5.03E-08	-1.68E-03	2.79E-03	5.49E-01	Cholelithiasis in the FinnGen Biobank	Concentration of large HDL particles || id: ebi-a-GCST90092851	73	-8.10E-02	3.19E-02	1.11E-02	3.36E-02	-3.43E-03	2.65E-03	2.00E-01	1.76E-02	4.11%
Triglyceride levels	31	Triglyceride levels in IDL || id: ebi-a-GCST90092841	Body mass index || id: ieu-a-835	68	7.71E-02	2.64E-02	3.51E-03	5.94E-03	1.71E-03	2.12E-03	4.23E-01	Cholelithiasis in the FinnGen Biobank	Triglyceride levels in IDL || id: ebi-a-GCST90092841	51	-8.75E-02	3.38E-02	9.61E-03	3.19E-02	3.84E-03	3.25E-03	2.44E-01	-6.74E-03	-1.58%
	32	Triglyceride levels in large LDL || id: ebi-a-GCST90092866	Body mass index || id: ieu-a-835	68	7.86E-02	2.60E-02	2.53E-03	4.46E-03	1.30E-03	2.10E-03	5.38E-01	Cholelithiasis in the FinnGen Biobank	Triglyceride levels in large LDL || id: ebi-a-GCST90092866	49	-1.14E-01	3.65E-02	1.81E-03	1.17E-02	1.48E-03	3.33E-03	6.59E-01	-8.95E-03	-2.09%
	33	Triglycerides to total lipids ratio in medium VLDL || id: ebi-a-GCST90092927	Body mass index || id: ieu-a-835	67	1.21E-01	3.46E-02	4.41E-04	1.05E-03	3.28E-03	2.74E-03	2.36E-01	Cholelithiasis in the FinnGen Biobank	Triglycerides to total lipids ratio in medium VLDL || id: ebi-a-GCST90092927	60	1.71E-01	4.37E-02	8.80E-05	1.58E-03	1.22E-03	3.49E-03	7.27E-01	2.08E-02	4.87%
	34	Triglycerides to total lipids ratio in small VLDL || id: ebi-a-GCST90092979	Body mass index || id: ieu-a-835	67	9.17E-02	3.16E-02	3.74E-03	6.26E-03	2.43E-03	2.52E-03	3.39E-01	Cholelithiasis in the FinnGen Biobank	Triglycerides to total lipids ratio in small VLDL || id: ebi-a-GCST90092979	49	1.11E-01	4.21E-02	8.46E-03	2.88E-02	-6.46E-03	3.86E-03	1.01E-01	1.02E-02	2.38%
	35	Triglycerides to total lipids ratio in very large VLDL || id: ebi-a-GCST90093027	Body mass index || id: ieu-a-835	67	9.83E-02	2.98E-02	9.74E-04	1.98E-03	4.00E-03	2.34E-03	9.24E-02	Cholelithiasis in the FinnGen Biobank	Triglycerides to total lipids ratio in very large VLDL || id: ebi-a-GCST90093027	42	1.33E-01	5.26E-02	1.17E-02	3.41E-02	-2.05E-03	5.39E-03	7.06E-01	1.30E-02	3.05%
Phospholipid levels	36	Phospholipid levels in IDL || id: ebi-a-GCST90092839	Body mass index || id: ieu-a-835	67	-9.77E-02	3.20E-02	2.28E-03	4.11E-03	5.36E-04	2.57E-03	8.35E-01	Cholelithiasis in the FinnGen Biobank	Phospholipid levels in IDL || id: ebi-a-GCST90092839	45	-1.12E-01	3.86E-02	3.77E-03	1.74E-02	5.01E-03	3.80E-03	1.94E-01	1.09E-02	2.55%
	37	Phospholipids to total lipids ratio in large HDL || id: ebi-a-GCST90092853	Body mass index || id: ieu-a-835	66	2.29E-01	3.83E-02	2.20E-09	6.69E-08	4.76E-03	2.99E-03	1.17E-01	Cholelithiasis in the FinnGen Biobank	Phospholipids to total lipids ratio in large HDL || id: ebi-a-GCST90092853	61	1.33E-01	4.64E-02	4.05E-03	1.78E-02	5.35E-04	4.00E-03	8.94E-01	3.06E-02	7.14%
	38	Phospholipid levels in large LDL || id: ebi-a-GCST90092864	Body mass index || id: ieu-a-835	67	-7.28E-02	3.07E-02	1.78E-02	2.61E-02	7.67E-04	2.46E-03	7.57E-01	Cholelithiasis in the FinnGen Biobank	Phospholipid levels in large LDL || id: ebi-a-GCST90092864	36	-1.14E-01	4.28E-02	7.94E-03	2.75E-02	2.43E-03	4.39E-03	5.83E-01	8.27E-03	1.93%
	39	Phospholipids to total lipids ratio in medium VLDL || id: ebi-a-GCST90092925	Body mass index || id: ieu-a-835	67	-9.61E-02	3.15E-02	2.29E-03	4.11E-03	-2.31E-03	2.51E-03	3.62E-01	Cholelithiasis in the FinnGen Biobank	Phospholipids to total lipids ratio in medium VLDL || id: ebi-a-GCST90092925	44	-1.27E-01	4.11E-02	1.93E-03	1.18E-02	3.18E-03	4.27E-03	4.61E-01	1.22E-02	2.86%
	40	Phospholipids to total lipids ratio in small VLDL || id: ebi-a-GCST90092977	Body mass index || id: ieu-a-835	66	-1.39E-01	3.18E-02	1.19E-05	5.39E-05	-1.80E-03	2.52E-03	4.79E-01	Cholelithiasis in the FinnGen Biobank	Phospholipids to total lipids ratio in small VLDL || id: ebi-a-GCST90092977	53	-1.13E-01	4.13E-02	6.45E-03	2.43E-02	4.05E-03	3.62E-03	2.68E-01	1.57E-02	3.66%
	41	Phospholipid levels in VLDL || id: ebi-a-GCST90093001	Body mass index || id: ieu-a-835	68	6.56E-02	2.74E-02	1.69E-02	2.49E-02	1.57E-03	2.21E-03	4.80E-01	Cholelithiasis in the FinnGen Biobank	Phospholipid levels in VLDL || id: ebi-a-GCST90093001	37	-1.19E-01	4.84E-02	1.37E-02	3.84E-02	4.62E-04	5.20E-03	9.30E-01	-7.82E-03	-1.83%
	42	Sphingomyelin levels || id: ebi-a-GCST90092982	Body mass index || id: ieu-a-835	65	-1.13E-01	3.42E-02	9.76E-04	1.98E-03	-3.56E-04	2.73E-03	8.97E-01	Cholelithiasis in the FinnGen Biobank	Sphingomyelin levels || id: ebi-a-GCST90092982	51	-1.21E-01	3.72E-02	1.11E-03	8.64E-03	5.22E-03	3.68E-03	1.63E-01	1.37E-02	3.20%
Total lipid levels	43	Total lipid levels in IDL || id: ebi-a-GCST90092837	Body mass index || id: ieu-a-835	67	-9.99E-02	3.25E-02	2.14E-03	3.90E-03	1.02E-03	2.61E-03	6.98E-01	Cholelithiasis in the FinnGen Biobank	Total lipid levels in IDL || id: ebi-a-GCST90092837	46	-1.06E-01	4.11E-02	1.01E-02	3.29E-02	5.15E-03	4.24E-03	2.30E-01	1.06E-02	2.47%
	44	Total lipid levels in large LDL || id: ebi-a-GCST90092862	Body mass index || id: ieu-a-835	67	-7.75E-02	3.17E-02	1.45E-02	2.17E-02	4.48E-04	2.54E-03	8.61E-01	Cholelithiasis in the FinnGen Biobank	Total lipid levels in large LDL || id: ebi-a-GCST90092862	36	-1.27E-01	4.29E-02	3.08E-03	1.51E-02	1.15E-03	4.60E-03	8.04E-01	9.84E-03	2.30%
Fatty acid levels	45	Ratio of docosahexaenoic acid to total fatty acid levels || id: ebi-a-GCST90092817	Body mass index || id: ieu-a-835	68	-1.10E-01	2.66E-02	3.62E-05	1.41E-04	1.04E-04	2.15E-03	9.61E-01	Cholelithiasis in the FinnGen Biobank	Ratio of docosahexaenoic acid to total fatty acid levels || id: ebi-a-GCST90092817	20	-2.56E-01	6.87E-02	1.90E-04	2.63E-03	3.87E-03	7.25E-03	6.00E-01	2.81E-02	6.57%
	46	Omega-6 fatty acid levels || id: ebi-a-GCST90092933	Body mass index || id: ieu-a-835	66	-6.60E-02	2.80E-02	1.84E-02	2.68E-02	1.17E-03	2.23E-03	6.02E-01	Cholelithiasis in the FinnGen Biobank	Omega-6 fatty acid levels || id: ebi-a-GCST90092933	41	-1.42E-01	4.85E-02	3.44E-03	1.62E-02	6.49E-03	5.47E-03	2.43E-01	9.36E-03	2.19%
	47	Linoleic acid levels || id: ebi-a-GCST90092880	Body mass index || id: ieu-a-835	66	-7.86E-02	2.64E-02	2.91E-03	4.99E-03	-1.70E-05	2.10E-03	9.94E-01	Cholelithiasis in the FinnGen Biobank	Linoleic acid levels || id: ebi-a-GCST90092880	32	-1.62E-01	5.46E-02	2.98E-03	1.51E-02	1.27E-02	6.62E-03	6.44E-02	1.27E-02	2.98%
	48	Degree of unsaturation || id: ebi-a-GCST90092994	Body mass index || id: ieu-a-835	68	-1.16E-01	2.92E-02	7.23E-05	2.43E-04	-5.31E-04	2.36E-03	8.23E-01	Cholelithiasis in the FinnGen Biobank	Degree of unsaturation || id: ebi-a-GCST90092994	27	-2.20E-01	6.68E-02	9.93E-04	8.38E-03	-4.23E-03	6.58E-03	5.26E-01	2.55E-02	5.95%
Other Metabolites	49	Ratio of apolipoprotein B to apolipoprotein A1 levels || id: ebi-a-GCST90092810	Body mass index || id: ieu-a-835	68	8.07E-02	3.22E-02	1.21E-02	1.88E-02	1.31E-04	2.60E-03	9.60E-01	Cholelithiasis in the FinnGen Biobank	Ratio of apolipoprotein B to apolipoprotein A1 levels || id: ebi-a-GCST90092810	53	-1.26E-01	3.07E-02	4.14E-05	1.47E-03	4.85E-03	2.84E-03	9.39E-02	-1.02E-02	-2.38%

The left part of the table displays the effect of body mass index on potential mediators (IVW method) and the results of horizontal pleiotropy. The right part of the table shows the effect of potential mediators on cholelithiasis (IVW method) and the results of horizontal pleiotropy. The MR-Egger method is employed for detecting horizontal pleiotropy due to its allowance for the presence of non-zero intercepts (The columns in the table corresponding to “egger_intercept”, “se”, and “pval”), a pval below 0.05 suggests the existence of horizontal pleiotropy. The mediation effect is calculated as the product of the beta coefficient for the association between body mass index and mediators and the beta coefficient for the association between mediators and cholelithiasis. The mediation effect refers to the effect of body mass index on cholelithiasis mediated by these mediators, while the direct effect refers to the direct impact of body mass index on cholelithiasis (total effect). IVW: Inverse Variance Weighted method; SNPs: Single-nucleotide polymorphisms; q_value: The P value post FDR method (Benjamini and Hochberg) calibration.

### The impact of BMI on amino acids and cholelithiasis risk

This study included nine individual amino acids and one group of branched-chain amino acids, which are composed of multiple amino acids. As shown in Table 2, we found that BMI is associated with 8 of them, including phenylalanine levels (β = 0.116, *p* < 0.0001), glutamine levels (β= -0.100, *p* = 0.00237), glycine levels (β= -0.136, *p* < 0.0001), isoleucine levels (β = 0.118, *p* < 0.0001), total branched-chain amino acids (β = 0.150, *p* < 0.0001), leucine levels (β = 0.125, *p* < 0.0001), tyrosine levels (β = 0.153, *p* < 0.0001), and valine levels (β = 0.166, *p* < 0.0001). More information can be found in Table 2. As presented in Table 3, our analysis indicates a significant positive correlation between alanine levels and the risk of developing cholelithiasis (OR = 1.22, *p* = 0.00309). Although BMI affects amino acid levels, we found that this change has no impact on the risk of cholelithiasis.

### The impact of BMI on cholesteryl ester levels and cholelithiasis risk

As presented in Table 2, we identified a significant association between BMI and 21 out of the 32 absolute and relative cholesterol ester levels included in our study. As demonstrated in Table 3, our study identified 15 cholesteryl esters levels associated with cholelithiasis from the FinnGen cohort. Additionally, as shown in Table 4, further analysis indicated that 10 cholesterol ester levels mediated the relationship between BMI and cholelithiasis risk, with the mediated proportion of risk ranging from 2.29 to 5.48%.

### The impact of BMI on free cholesterol levels and cholelithiasis risk

As presented in Table 2, we identified a significant association between BMI and 26 out of the 32 absolute and relative free cholesterol levels included in our study. As demonstrated in Table 3, our study found 14 free cholesterol levels to be associated with cholelithiasis from the FinnGen cohort. Additionally, as shown in Table 4, further analysis indicated that 9 free cholesterol levels mediated the relationship between BMI and cholelithiasis risk, with the mediated proportion of risk ranging from 2.23 to 4.36%.

### The impact of BMI on cholesterol levels and cholelithiasis risk

As presented in Table 2, we identified a significant association between BMI and 27 out of the 35 absolute and relative cholesterol Levels included in our study. As demonstrated in Table 3, our study found that 17 cholesterol levels are associated with cholelithiasis from the FinnGen cohort. Additionally, as shown in Table 4, further analysis indicated that 10 cholesterol Levels mediated the relationship between BMI and cholelithiasis risk, with the mediated proportion of risk ranging from 1.69 to 4.77%.

### The impact of BMI on particle concentrations or sizes and cholelithiasis risk

As shown in Table 2, we identified a significant association between BMI and 12 out of three different particle sizes, as well as 18 absolute or relative particle concentrations included in our study. As demonstrated in Table 3, our study found that 6 particle concentrations are associated with cholelithiasis from the FinnGen cohort. Furthermore, Table 4 reveals that only the concentration of large HDL particles mediated the relationship between BMI and cholelithiasis risk, with a mediation proportion of 4.11%.

### The impact of BMI on triglyceride levels and cholelithiasis risk

As presented in Table 2, we identified a significant association between BMI and 28 out of the 32 absolute and relative triglyceride levels included in our study. As demonstrated in Table 3, our study found that 6 triglyceride levels are associated with cholelithiasis from the FinnGen cohort. Additionally, as shown in Table 4, further analysis indicated that 5 triglyceride levels mediated the relationship between BMI and cholelithiasis risk, with the mediated proportion of risk ranging from − 2.09 to 4.87%.

### The impact of BMI on phospholipid levels and cholelithiasis risk

As presented in Table 2, we identified a significant association between BMI and 24 out of the 37 absolute and relative phospholipid levels included in our study. As demonstrated in Table 3, our study found that 14 phospholipid levels are associated with cholelithiasis from the FinnGen cohort. Additionally, as shown in Table 4, further analysis indicated that 7 phospholipid levels mediated the relationship between BMI and cholelithiasis risk, with the mediated proportion of risk ranging from − 1.83 to 7.14%.

### The impact of BMI on total lipid levels and cholelithiasis risk

As presented in Table 2, we identified a significant association between BMI and 11 out of the 18 absolute and relative total lipid levels included in our study. As demonstrated in Table 3, our study found that 6 total lipid levels are associated with cholelithiasis from the FinnGen cohort. Additionally, as shown in Table 4, further analysis indicated that 2 total lipid levels mediated the relationship between BMI and cholelithiasis risk, with the mediated proportion of risk ranging from 2.30 to 2.47%.

### The impact of BMI on fatty acid levels and cholelithiasis risk

As presented in Table 2, we identified a significant association between BMI and 12 out of the 18 absolute and relative fatty acid levels included in our study. As demonstrated in Table 3, our study found that 4 fatty acid levels are associated with cholelithiasis from the FinnGen cohort. Additionally, as shown in Table 4, further analysis indicated that 4 fatty acid levels mediated the relationship between BMI and cholelithiasis risk, with the mediated proportion of risk ranging from 2.19 to 6.57%.

### The impact of BMI on other metabolites and cholelithiasis risk

The study also included 14 additional metabolites, consisting of three apolipoproteins, four metabolites related to glycolysis, glycoprotein acetyls, two ketone bodies, acetate, acetone, albumin, and creatinine. As shown in Table 2, we found that BMI was associated with acetoacetate levels (β = 0.0926, *p* = 0.000486), albumin levels (β=-0.107, *p* = 0.000134), apolipoprotein A1 levels (β=-0.161, *p* < 0.0001), the ratio of apolipoprotein B to apolipoprotein A1 levels (β = 0.0807, *p* = 0.0121), glucose levels (β = 0.0940, *p* = 0.000153), glycoprotein acetyls levels (β = 0.166, *p* < 0.0001), and 3-hydroxybutyrate levels (β = 0.0648, *p* = 0.0300). As demonstrated in Table 3, our study found that 2 other metabolite levels are associated with cholelithiasis from the FinnGen cohort. Additionally, as shown in Table 4, further analysis indicated that the ratio of apolipoprotein B to apolipoprotein A1 levels mediated the relationship between BMI and cholelithiasis risk, with the mediated proportion of -2.38%.

## Discussion

This study highlights the significant association between BMI and various metabolic biomarkers, providing insight into how BMI influences metabolic processes and the risk of cholelithiasis. The analysis identified 176 metabolites associated with BMI, of which 49 were found to mediate the relationship between BMI and cholelithiasis, indicating the complex pathways through which BMI impacts disease risk.

Our findings reveal a significant association between BMI and several key metabolic traits, including amino acids, cholesterol, and lipoproteins. For instance, both our study and previous research^20^ have demonstrated that BMI is associated with increased levels of branched-chain amino acids. These amino acids have previously been linked to metabolic disorders, including insulin resistance^21,22^, cardiovascular diseases^23,24^, and type 2 diabetes^20^, suggesting that higher BMI may exacerbate metabolic dysfunction through amino acid metabolism. However, despite the significant changes in amino acid levels, our analysis did not find evidence that these shifts contribute directly to cholelithiasis risk. Cholesterol metabolism also emerged as a critical pathway influenced by BMI. We observed significant associations between BMI and both cholesteryl ester and free cholesterol levels. A previous study^10^ revealed a negative correlation between BMI and serum HDL cholesterol levels, while another study showed an inverse relationship between HDL cholesterol and the risk of cholelithiasis^25^. This seems to suggest a potential pathway through which BMI may influence the risk of cholelithiasis, and our results also support this conclusion. Notably, several cholesterol esters and free cholesterol levels mediated the relationship between BMI and cholelithiasis, with mediation proportions ranging from 2 to 5%. This suggests that BMI-related alterations in cholesterol metabolism could play a role in the development of cholelithiasis, particularly through increased cholesterol saturation in bile. Additionally, BMI was found to influence several other metabolic markers, including acetoacetate, glucose, and glycoprotein acetyls. These markers are indicative of broader metabolic changes associated with obesity, such as impaired glucose metabolism and inflammation. Notably, the ratio of apolipoprotein B to apolipoprotein A1, an important marker of cardiovascular risk^26^, was also affected by BMI and mediated the relationship between BMI and cholelithiasis, although the mediated effect was negative (-2.38%). We found that as BMI increases, this ratio also increases. However, we found that this higher ratio is inversely associated with the risk of cholelithiasis. The negative mediation effects observed for the ratio of apolipoprotein B to apolipoprotein A1, phospholipid levels in VLDL, and triglyceride levels in large LDL and IDL suggest a complex interplay between lipid metabolism, BMI, and cholelithiasis risk. While BMI is generally associated with increased risk of cholelithiasis through lipid dysregulation, these reverse mediation effects imply that certain lipid profile changes may counteract or modulate this risk. For example, alterations in the distribution of apolipoproteins or triglycerides may influence cholesterol saturation in bile, potentially reducing the risk of gallstone formation despite the overall increase in BMI. These findings highlight the nuanced role of lipid metabolism in the BMI-cholelithiasis relationship and suggest that not all lipid changes driven by BMI necessarily exacerbate gallstone risk.

Our study reveals that BMI influences the risk of cholelithiasis through multiple metabolic pathways, particularly in lipid metabolism. First, BMI is significantly associated with various cholesterol-related metabolites, such as cholesterol esters and free cholesterol. These changes may promote gallstone formation by influencing the saturation of cholesterol in bile. Other metabolites, such as fatty acids and phospholipids, also participate in this process. The levels of these metabolites change with increasing BMI and may affect the risk of cholelithiasis by altering lipid accumulation in the liver and gallbladder. Furthermore, although amino acid metabolism changes are closely linked to BMI, our analysis did not find direct evidence that amino acid level changes contribute to gallstone risk. Overall, the metabolic changes triggered by BMI affect the occurrence of gallstones through various pathways, such as lipid metabolism, suggesting that controlling BMI may help reduce metabolic dysfunctions and, thereby, lower the risk of cholelithiasis.

This study inevitably has certain limitations. First, the findings cannot be generalized to populations outside of Europe, as the data used primarily came from European populations. Second, due to data limitations, we were unable to perform subgroup analyses based on age and gender. Third, also due to data constraints, while the study provides qualitative insights, it cannot conclusively demonstrate whether certain metabolic levels increase and then decrease as BMI rises.

## Conclusion

This study emphasizes the role of BMI in altering key metabolic pathways, including cholesterol, triglyceride, phospholipid, and fatty acid metabolism, which contribute to gallstone formation. We identified metabolites such as cholesteryl esters, free cholesterol, triglycerides, and phospholipids, which mediate the BMI-cholelithiasis relationship. These findings reinforce the critical role of BMI in shaping metabolic profiles, suggesting that controlling BMI may help mitigate disruptions in metabolic processes and reduce disease risk. Future research should focus on further understanding these mechanisms and exploring potential therapeutic targets.

## Electronic Supplementary Material

Below is the link to the electronic supplementary material.

Supplementary Material 1

## Data Availability

All GWAS data used in this study are available in the IEU open GWAS project (https://gwas.mrcieu.ac.uk/) and the FinnGen Biobank (https://r10.risteys.finngen.fi/).
